# Transformation of enriched mammary cell populations with polyomavirus middle T antigen influences tumor subtype and metastatic potential

**DOI:** 10.1186/s13058-015-0641-9

**Published:** 2015-10-01

**Authors:** Daria Drobysheva, Brittni Alise Smith, Maria McDowell, Katrin P. Guillen, Huseyin Atakan Ekiz, Bryan E. Welm

**Affiliations:** Department of Oncological Sciences, University of Utah, 315 South 1400 East, Salt Lake City, UT 84112 USA; Immunobiology and Cancer Program, Oklahoma Medical Research Foundation, 825 Northeast 13th Street, Oklahoma City, OK 73104 USA; Current address: Department of Surgery, Huntsman Cancer Institute, University of Utah, 2000 Circle of Hope Drive, Salt Lake City, UT 84112 USA

## Abstract

**Introduction:**

Breast cancer exhibits significant molecular, histological, and pathological diversity. Factors that impact this heterogeneity are poorly understood; however, transformation of distinct normal cell populations of the breast may generate different tumor phenotypes. Our previous study demonstrated that the polyomavirus middle T antigen (*PyMT*) oncogene can establish diverse tumor subtypes when broadly expressed within mouse mammary epithelial cells. In the present study, we assessed the molecular, histological, and metastatic outcomes in distinct mammary cell populations transformed with the *PyMT *gene.

**Methods:**

Isolated mouse mammary epithelial cells were transduced with a lentivirus encoding *PyMT* during an overnight infection and then sorted into hormone receptor–positive luminal (CD133+), hormone receptor–negative luminal (CD133−), basal, and stem cell populations using the cell surface markers CD24, CD49f, and CD133. Each population was subsequently transplanted into syngeneic cleared mouse mammary fat pads to generate tumors. Tumors were classified by histology, estrogen receptor status, molecular subtype, and metastatic potential to investigate whether transformation of different enriched populations affects tumor phenotype.

**Results:**

Although enriched mammary epithelial cell populations showed no difference in either the ability to form tumors or tumor latency, differences in prevalence of solid adenocarcinomas and squamous, papillary, and sebaceous-like tumors were observed. In particular, squamous metaplasia was observed more frequently in tumors derived from basal and stem cells than in luminal cells. Interestingly, both molecularly basal and luminal tumors developed from luminal CD133+, basal, and stem cell populations; however, luminal CD133− cells gave rise exclusively to molecularly basal tumors. Tumors arising from the luminal CD133−, basal, and stem cell populations were highly metastatic; however, luminal CD133+ cells generated tumors that were significantly less metastatic, possibly due to an inability of these tumor cells to escape the primary tumor site.

**Conclusions:**

Expression of *PyMT* within different mammary cell populations influences tumor histology, molecular subtype, and metastatic potential. The data demonstrate that luminal CD133+ cells give rise to less metastatic tumors, luminal CD133− cells preferentially establish basal tumors, and the cell of origin for squamous metaplasia likely resides in the basal and stem cell populations.

**Electronic supplementary material:**

The online version of this article (doi:10.1186/s13058-015-0641-9) contains supplementary material, which is available to authorized users.

## Introduction

The classification of breast cancer into several distinct molecular and histological subtypes can provide information to help guide patient therapy and predict outcome [1, 2]. Tumors that retain histological and molecular attributes of normal tissue are considered well differentiated and are generally less aggressive and correlate with better patient prognosis. In contrast, the loss of normal tissue structure and the dysregulation of genes involved in modulating growth and differentiation indicate transition of the disease into a more advanced stage [3]. A better understanding of tumor etiology and processes that control the transition between early and advanced states of breast cancer may improve strategies for detection, treatment, and prevention of the disease.

How a particular cell responds to a transforming event, its susceptibility for malignant progression, and its role in establishing a tumor’s histological and molecular fate are poorly understood. The mammary gland is a complex tissue composed of two distinct cell lineages, the luminal epithelium and myoepithelium, with each lineage encompassing a hierarchy of cells at various states of differentiation [4, 5]. When a normal cell is transformed, preexisting signaling networks intrinsic to that particular cell type may become dysregulated and contribute to tumor growth and progression. For example, tumors classified as a hormone-receptor positive subtype express estrogen receptor (ER or* Esr1*) and are generally dependent on estrogen for growth, recapitulating characteristics of a subset of normal, luminal epithelial cells found in the breast [6]. Molecular similarities are also observed between other normal cell populations and cancer subtypes. Mammary stem cells have a gene expression signature similar to spindloid and claudin-low tumors [7–9], whereas the molecular signature of normal luminal progenitors is associated with basal-like breast cancer [8]. Even differentiated mammary epithelial cells (MECs) share molecular features with a cancer subtype. Tumors histologically classified as lipid-rich carcinoma of the breast express metabolic and differentiation markers observed in alveolar cells, the milk-producing cells of the mammary gland [10–12]. These molecular associations suggest that tumors can arise from different cell populations and maintain signaling networks of their cell of origin.

Although transforming events within a cell may initiate and drive the process of tumor progression, these events do not necessarily establish a tumor’s histological or molecular fate. Data derived from The Cancer Genome Atlas demonstrate that major oncogenic drivers and tumor-associated mutations are broadly represented among breast cancer subtypes, suggesting that diverse mechanisms contribute to a tumor’s phenotype [13]. Such mechanisms may include cell-intrinsic networks acquired from the cell of origin. In support of this, several studies have demonstrated that normal cell types can respond to the same oncogenic pathway in unique ways. Ince et al. used different cell culture conditions to enrich for either luminal-like or myoepithelial-like human breast cell populations [14]. Both populations were subsequently transformed with a common set of oncogenic drivers. When transplanted, each precursor cell population generated a distinct tumor phenotype, with notable differences in tumor histology and metastasis. The myoepithelial-like cells established tumors similar to squamous carcinoma of the breast, whereas the luminal-like cells generated papillary adenocarcinomas [14]. Other studies with mouse mammary tumor models have also supported a role for the cell of origin in controlling tumor fate. Differences in tumor phenotype were observed when a *Brca1* mutation was induced in different normal cell populations. Conditional *Brca1* loss of function targeted to the basal compartment using *Keratin-14-Cre Brca1*^*fl/fl*^*p53*^*+/−*^ mice established tumors that were primarily adenosquamous carcinomas and adenomyoepitheliomas. In contrast, disruption of *Brca1* in luminal progenitors using *Blg-Cre Brca1*^*fl/fl*^*p53*^*+/−*^ mice resulted in ductal carcinomas with a basal-like molecular subtype [15]. These studies suggest that intrinsic differences between cell populations may influence the histopathology of the tumors they generate.

The polyomavirus middle T antigen (*PyMT*) oncogene has been used extensively in mice to model breast cancer [16, 17]. In these models, *PyMT* drives transformation of MECs by signaling through several pathways, including *Src*, *Ras*, and phosphoinositide 3-kinase [18–21], resulting in a phenotype similar to ErbB2/Neu-induced tumors [7, 22, 23]. Transgenic mice that express *PyMT* under control of the promoter derived from the long terminal repeat (LTR) of the mouse mammary tumor virus (MMTV) develop mammary tumors that undergo progressive transition from precancerous lesions to late-stage malignant tumors and exhibit a high frequency of metastasis [16, 24, 25]. Tumor progression is marked by a loss of both myoepithelial cells and ER+ luminal cells [24], and a concomitant expansion of cells expressing the luminal progenitor marker CD61 [26]. Through intrinsic gene set analysis and hierarchical clustering of gene expression profiles, MMTV-*PyMT* tumors have been classified within the luminal subgroup [7, 9]. In addition, a close association between the molecular signature derived from luminal progenitors and MMTV-*PyMT* tumors has been described [9]. Similar to other mouse models that function through ErbB/Ras signaling proteins, the predominant tumor histology in the MMTV-*PyMT* model is solid adenocarcinoma. However, varied histopathology is observed, with approximately 30 % of tumors having papillary, glandular, or acinar features and 10 % exhibiting either adenosquamous, pilar, or type P histology [22].

These data demonstrate that the MMTV-*PyMT* model can establish tumors with both a luminal phenotype and diverse histopathology. It is unknown whether these characteristics are a result of the activity of the MMTV LTR within a particular cell type or through unique transforming activity of the PyMT oncogene. Because the MMTV LTR is widely active in mammary epithelium and drives expression at early stages of postnatal development [27–29], it is difficult to identify the cell of origin for tumors in this model. In order to assess how cellular context affects tumor progression, the *PyMT* oncogene has been targeted to various MEC populations by uncoupling expression of the oncogene from the MMTV LTR. For example, virus-based approaches have been used to express* PyMT *either ubiquitously in all mammary cell populations or specifically within distinct cell types, and these studies have shown that tumor histology and molecular subtype can vary as a result of the targeting approach [12, 30, 31]. The restricted expression of *PyMT* in the keratin 6 (K6) mammary cell population resulted in tumors with predominantly papillary and cystic histology and a distinct normal-like molecular subtype, representing a phenotype dissimilar to what has been observed in the MMTV-*PyMT* mouse model [27]. In contrast, non-cell-type–specific expression of *PyMT* in MECs using the ubiquitous elongation factor 1 alpha (EF1α) promoter generated tumors with diverse histology, including a high frequency of adenosquamous carcinomas and the occurrence of a unique lipid-rich carcinoma [12]. In addition, this model produced tumors that classified within both luminal and basal molecular subgroups [12]. In the present study, we extended upon these studies by evaluating the latency, histopathology, molecular subgroup, and metastatic potential of tumors derived from four different fluorescence-activated cell sorting (FACS)-enriched MEC populations. The results suggest that the originating cell population influences several tumor characteristics and further implicates a cell residing predominantly in the basal and stem cell compartment as the cellular origin for squamous metaplasia.

## Methods

### Mice

FVB/NJ mice were obtained from The Jackson Laboratory (Bar Harbor, ME, USA) and maintained in a pathogen-free facility. The University of Utah Institutional Animal Care and Use Committee approved mouse handling and procedures.

### Generation of mouse mammary tumors

MECs were collected from 8–10-week-old FVB/NJ mice as described previously [12]. Then, freshly isolated MECs were infected with EF1α-*PyMT-ZsGreen *lentivirus overnight at 37 °C as described previously [12]. Following infection, cells were washed five times with Hanks’ balanced salt solution (HBSS; Gibco/Thermo Fisher Scientific, Grand Island, NY, USA) and incubated with 0.05 % trypsin-ethylenediaminetetraacetic acid (EDTA; Gibco/Thermo Fisher Scientific, Grand Island, NY, USA) to isolate single cells. Trypsin was inactivated with MEC media [12], and cell clumps were removed by straining MECs through a 40-μm cell strainer (Falcon; Fisher Scientific, Pittsburgh, PA, USA). MECs were then resuspended in wash buffer (HBSS + 2 % fetal bovine serum; HyClone Laboratories/GE Healthcare, Logan, UT, USA) and kept on ice for antibody staining and FACS.

Staining consisted of six tubes: (1) no antibody control, (2) CD24-V450 control, (3) CD49f-phycoerythrin (CD49f-PE) control, (4) CD133-allophycocyanin (CD133-APC) control, (5) 7-amino-actinomycin D (7-AAD) control, and (6) CD24-V450/CD49f-PE/CD133-APC/7-AAD sample. During antibody staining, control tubes contained 5 × 10^4^ cells and the sample tube contained 20 × 10^6^ cells resuspended in 200 μl of wash buffer. All antibodies were obtained from BD Pharmingen (San Diego, CA, USA) and were used at a 1:100 dilution. After the primary antibodies were added, cells were incubated on ice for 15 minutes. Following incubation, cells were washed with 1 ml of wash buffer and centrifuged at 1000 × *g* for 2 minutes. Stained MECs were then resuspended in wash buffer and sorted into luminal CD133+, luminal CD133−, basal, and stem cell populations as described previously [32–35] on a BD FACSAria Cell Sorter using FACSDiva version 6.1.3 software for analysis (BD Biosciences, San Jose, CA, USA). Isolated MEC populations were kept on ice until transplantation.

For each transplantation, 1 × 10^5^ untransduced and unsorted MECs were mixed with 2 × 10^4^ transduced luminal CD133+, luminal CD133−, basal, or 5 × 10^3^ stem enriched MECs. MECs were then resuspended in 10 μl of Matrigel (BD Biosciences, San Jose, CA, USA) by transplantation, and the Matrigel cell mixture was injected into the fourth cleared inguinal mammary fat pad of 3-week-old FVB/NJ mice. Only a single fat pad was injected per mouse. Tumor growth was monitored, and tumors were collected upon reaching 2 cm in diameter. Once tumors were harvested, viable cells were collected using the same protocol for MEC isolation and then frozen in freeze media as described previously [12]. Portions of the tumors were also flash-frozen for RNA isolation using a Qiagen RNeasy kit (Qiagen, Valencia, CA, USA), and additional tumor fragments were processed for paraffin embedding.

Infection, cell sorting, and transplantation experiments were performed over two rounds. Each time, 10 transplants were performed per sorted MEC population, for a total 20 transplants per group.

### Antibody staining and histology

Portions of transduced and FACS-sorted MECs were used to quantify basal and luminal cell enrichment. For each isolated population, 1 × 10^4^ MECs resuspended in 200 μl of wash buffer were centrifuged (Cytospin 4 Cytocentrifuge; Thermo Scientific, Hudson, NH, USA) onto slides (Shandon Cytoslide; Thermo Scientific, Hudson, NH, USA) at 900 rpm for 10 minutes. Cytospun cells were then incubated with fixative solution (4 % paraformaldehyde in phosphate-buffered saline [PBS]) for 15 minutes and washed five times with PBS for 5 minutes each. Fixed cells were permeabilized for 10 minutes with 0.2 % Triton X-100 (Sigma-Aldrich, St. Louis, MO, USA) in PBS, washed with 1 % bovine serum albumin (BSA; EMD Millipore, Billerica, MA, USA) in PBS, and blocked with 1 % BSA in PBS for 10 minutes. Cells were then incubated with primary antibodies against keratin 14 (K14, 1:400 dilution, rabbit, PRB-P-100; Covance, Princeton, NJ, USA) and keratin 8 (K8, 1:50 dilution, rat, TROMA-I; Developmental Studies Hybridoma Bank, Iowa City, IA, USA) for 1 h at room temperature. Following incubation, slides were washed with 1 % BSA in PBS and stained with 4′,6-diamidino-2-phenylindole dihydrochloride and secondary antibodies Alexa Fluor 594 chicken anti-rat immunoglobulin G (IgG, 1:1000 dilution; Invitrogen/Life Technologies, Carlsbad, CA, USA) and Alexa Fluor 488 goat anti-rabbit IgG (1:1000 dilution; Invitrogen/Life Technologies, Carlsbad, CA, USA).

Paraffin-embedded tumor samples were processed and subjected to hematoxylin and eosin (H&E) staining, ESR1 immunohistochemistry (IHC), and cytokeratin staining as described previously [12]. The following primary antibodies were used: K14 (1:400 dilution, rabbit, PRB-P-100; Covance), K8 (1:50 dilution, rat, TROMA-I; Developmental Studies Hybridoma Bank, Iowa City, IA, USA), and ESR1 (1:200 dilution, MC-20, sc-542; Santa Cruz Biotechnology, Santa Cruz, CA, USA). Secondary antibodies included Alexa Fluor 594 chicken anti-rat IgG (1:1000 dilution; Invitrogen/Life Technologies, Carlsbad, CA, USA), Alexa Fluor 488 goat anti-rabbit IgG (1:1000 dilution; Invitrogen/Life Technologies, Carlsbad, CA, USA), and biotin-SP-conjugated protein (1:1000 dilution; Jackson ImmunoResearch Laboratories, West Grove, PA, USA).

All immunofluorescence imaging was performed on an Olympus IX81 microscope (Olympus, Tokyo, Japan) using a Hamamatsu Photonics ORCA-ER camera (Hamamatsu Photonics, Hamamatsu City, Japan). Fluorescence image recording and processing were performed using SlideBook 64 version 5.0.0.24 software (Intelligent Imaging Innovations, Denver, CO, USA). Slides processed for IHC and H&E staining were imaged on an Olympus BX50 microscope with a Canon EOS Rebel XSI camera using EOS imaging software (Canon, Melville, NY, USA). Any changes in contrast and brightness were performed using Photoshop CS4 software (Adobe Systems, San Jose, CA, USA) on entire images to enhance appearance without altering image content.

### In vitro and in vivo assessment of tumor growth with estrogen receptor inhibition

For in vitro dose–response studies, tumor cells and MECs were isolated using the same tissue dissociation protocol as described above. Single cells were suspended in Matrigel (BD Biosciences, San Jose, CA, USA) and 10 μl of a cell/Matrigel mixture was plated per well in a 96-well plate (Costar; Corning Life Sciences, Oneonta, NY, USA). 4-Hydroxytamoxifen (Sigma-Aldrich, St. Louis, MO, USA) was dissolved in ethanol and serially diluted in MEC media. Cells were dosed with 100 μl of media containing drug or vehicle control for 48 h. Media with drug or vehicle control was refreshed every 24 h. Each drug concentration was tested in triplicate. Cell viability was measured using the ATPlite assay (PerkinElmer, Waltham, MA, USA) according to the manufacturer’s instructions and normalized to vehicle control.

Tumor growth dependence on estrogen was tested in vivo by conducting an additional primary cell infection, FACS, and transplantation experiments as described above. Ovaries were removed from all mice that received cell transplants, as described previously [36]. Ten surgeries were performed per infected and sorted population.

### Microarray analysis

Flash-frozen tumors were randomly selected from each of the tumor groups for RNA extraction and microarray analysis. Total RNA was isolated using the Qiagen RNeasy kit.

To perform supervised hierarchical clustering, all steps of microarray processing, data filtering and normalization, and analysis were performed as described previously [12]. Batch adjustment was performed in two batches. A dataset generated by Herschkowitz et al. was treated as one batch (Gene Expression Omnibus [GEO] accession number [GEO:GSE3165]) [7], and data generated at the Huntsman Cancer Institute (HCI) was treated as a second batch. The HCI microarray dataset has been deposited in the National Center for Biotechnology Information GEO database under accession number [GEO:GSE64453].

To perform unsupervised hierarchical clustering, quantile-normalized, log-scaled microarray intensity data were hierarchically clustered in R using Ward’s method. The resulting dendrogram revealed a marked batch effect related to the date on which the arrays were processed. Each class of samples (luminal CD133−, luminal CD133+, basal, and stem) were adjusted for batch effect separately using the ComBat procedure [37] and then recombined. Differential expression analysis comparing the luminal CD133− samples with the remaining samples identified 1111 microarray probes representing 1046 unique genes showing at least twofold differential expression at an adjusted *p* value <0.05 (*p* value by *t* test with adjustment using the Benjamini-Hochberg method).

### Assessment of tumor metastasis

As described above, transplantation experiments were performed twice for each transduced and enriched MEC population. Lung metastasis for the first round was assessed by H&E staining. Lung tissue processing and staining were performed as described above. Paraffin-embedded lungs were serially sectioned at 10 μm, and every fifth slide was stained and examined for metastasis. For the second round of transplants, lung metastases were analyzed by fluorescence imaging after lungs were flattened between two glass slides. Slides were imaged as described above, and numbers of unique metastatic sites and tumor areas were quantified using ImageJ software (National Institutes of Health, Bethesda, MD, USA). Prevalence of lung metastases and numbers of metastatic foci were consistent over two rounds of transplants.

To quantify circulating tumor cell (CTC) numbers, fresh whole blood was collected by cardiac puncture immediately after mice were killed according to University of Utah–approved Institutional Animal Care and Use Committee procedures. CTCs were isolated for FACS analysis as described previously from mice bearing primary tumors, as well as from no-tumor control mice [38]. CTCs expressing ZsGreen were detected by analyzing cells using a FACScan cytometer (BD Biosciences, San Jose, CA, USA), and results were quantified using FlowJo software (Tree Star, Ashland, OR, USA). Owing to low numbers of CTCs present within isolated whole blood, the ZsGreen-positive threshold was set at 0.05 % of no-tumor control background signal. This threshold was then used as a baseline for detecting CTCs in tumor-bearing mice. All CTC values were then normalized to no-tumor control background signal.

Tail vein injections were performed to assess the ability of tumor cells to colonize the lungs after introduction into the bloodstream. Single cells were isolated from primary tumors using the same procedure employed for MEC isolation. Cells were then resuspended in HBSS at 10 × 10^6^ cells/ml. A quantity of the HBSS/cell mixture (250 μl; 2.5 × 10^5^ cells) was injected into the lateral tail veins of 8–12-week-old FVB/NJ mice. Cells isolated from individual tumors were injected into five mice each. Twenty days postinjection, mice were killed and tumor lung foci numbers were quantified by fluorescence imaging with ImageJ software.

### Analysis of *PyMT* expression in transduced mammary epithelial cells

Freshly isolated MECs were infected in suspension overnight and then transferred to adherent culture in MEC media the following day. Separate plates of cells were analyzed for *ZsGreen* and *PyMT* expression each day for 5 days. Protein lysates were prepared for simple Western blot analysis to detect *PyMT* expression, and cells were analyzed for *ZsGreen* expression by flow cytometry. For cytometry, cells were washed with PBS, lifted with 0.25 % trypsin-EDTA (Gibco/Thermo Fisher Scientific), fixed in 2 % paraformaldehyde, and resuspended in wash buffer on ice. Single cells were isolated by straining them through a 40-μm cell strainer (Falcon; Fisher Scientific) and immediately analyzed with a BD LSR II flow cytometer (BD Biosciences, San Jose, CA, USA) for* ZsGreen *expression.

### Real-time quantitative PCR analysis

RNA was isolated from freshly sorted MECs or flash-frozen tumors using the Qiagen RNeasy Mini Kit. For cDNA synthesis from tumor RNA, 1 μg of total RNA was reverse-transcribed using the iScript Reverse Transcription Supermix for real-time quantitative PCR (RT-qPCR) synthesis kit (Bio-Rad Laboratories, Hercules, CA, USA) according to the manufacturer’s instructions. For sorted MECs, cDNA was synthesized from 20 ng of total RNA via reverse transcription, followed by preamplification with pooled PrimeTime assays (Integrated DNA Technologies, Coralville, IA, USA) for genes of interest using the Qiagen RT^2^ PreAMP cDNA Synthesis Kit. Genes investigated were *K8* (assay identification: Mm.PT.58.6862465), *K14* (Mm.PT.58.43652691), *progesterone receptor *(*Pgr*; Mm.PT.58.10254276), Esr1 (Mm.PT.58.8025728), *p63* (Mm.PT.58.13970687), *Slug* (Mm.PT.58.43645779), *c-Kit* (Mm.PT.56a.33701407), and *glyceraldehyde 3-phosphate dehydrogenase* (*Gapdh*; Mm.PT.39a.1) for reference. RT-qPCRs were performed in 20-μl volumes using 2× SYBR Green PCR Master Mix (Applied Biosystems, Foster City, CA, USA) in a Roche LightCycler96 system (Roche Applied Science, Indianapolis, IN, USA). The primers for *PyMT* and the reference gene, *Rplp0*, have been described previously [12]. Each sample was normalized to the reference gene, sorted MEC cycle threshold change (ΔC_T_) values were compared with a control population of unsorted MECs, and relative fold induction level for each sorted population/tumor was calculated using the 2^−ΔΔCt^ method [39].

### Simple Western blot analysis

Using a PowerGen 125 sawtooth homogenizer (Fisher Scientific), protein lysates were prepared from flash-frozen tumors using Pierce IP Lysis Buffer (25 mM Tris∙HCl, pH 7.4, 150 mM NaCl, 1 % Nonidet P-40, 1 mM EDTA, 5 % glycerol; Pierce Biotechnology/Thermo Scientific, Rockford, IL, USA) with 10 mM sodium pyrophosphate tetrabasic (Sigma-Aldrich, St. Louis, MO, USA), 1 mM sodium orthovanadate (Sigma-Aldrich, St. Louis, MO, USA), Phosphatase Inhibitor Cocktails 2 and 3 (Sigma-Aldrich, St. Louis, MO, USA), and Mammalian ProteaseArrest cocktail (G-Biosciences, St. Louis, MO, USA). Lysates from cultured cells were prepared without pyrophosphate tetrabasic and sodium orthovanadate. Protein was quantified using a DC Protein Assay Kit (Bio-Rad Laboratories, Hercules, CA, USA) and a DU 730 UV/VIS spectrophotometer (Beckman Coulter, Fullerton, CA, USA).

Simple Western blot analyses were performed as instructed by the ProteinSimple user manual. Briefly, cell lysates were mixed with a master mix (ProteinSimple, San Jose, CA, USA) to a final concentration of 1× sample buffer, 1× fluorescence molecular weight marker, and 40 nM dithiothreitol. Following 5-minute denaturation at 95 °C, the samples, blocking agent, primary antibodies, horseradish peroxidase–conjugated secondary antibody, and chemiluminescence substrate were dispensed into the designated wells of the 384-well plate. Automated separation electrophoresis and immunodetection were performed using a ProteinSimple WES instrument. All antibodies were diluted in the antibody diluent II (ProteinSimple, San Jose, CA, USA) and incubated with the protein for 10–15 minutes. A luminol-peroxide mixture (ProteinSimple, San Jose, CA, USA) was used to generate chemiluminescence, which was captured with a charge-coupled device camera. The resulting digital image was analyzed with Compass software (ProteinSimple, San Jose, CA, USA), and quantified data were reported as molecular weight, signal and peak intensity, and area under the curve.

The following antibodies used were obtained from Cell Signaling Technology (Danvers, MA, USA): β-actin (13E5, 1:50 dilution, catalog number 4970P), AKT (C67E7, 1:50 dilution, catalog number 4691P), extracellular signal-regulated kinase 1/2 (ERK1/2; 137 F5, 1:50 dilution, catalog number 4695P), SRC (36D10, 1:100 dilution, catalog number 2109S), phospho-AKT (Thr308, C31E5E, 1:25 dilution, catalog number 2965S), phospho-ERK1/2 (Thr202/Tyr204, dilution 1:50, catalog number 4370P), and phospho-SRC (Tyr527, 1:50 dilution, catalog number 2105P). The following antibodies were obtained from Santa Cruz Biotechnology(Santa Cruz, CA, USA): hemagglutinin probe (Y-11, 1:12.5 dilution, catalog number sc-805) and GAPDH (FL-335, 1:1500 dilution, catalog number sc-25778).

### Statistical analysis

All data analyses were performed in using GraphPad Prism 6.0d software (GraphPad Software, La Jolla, CA, USA). For each analysis, specific statistical tests are indicated in the figure legends.

## Results

### FACS-enriched mammary cell populations expressing *PyMT* develop tumors with equivalent latency

**Fig. 1:**
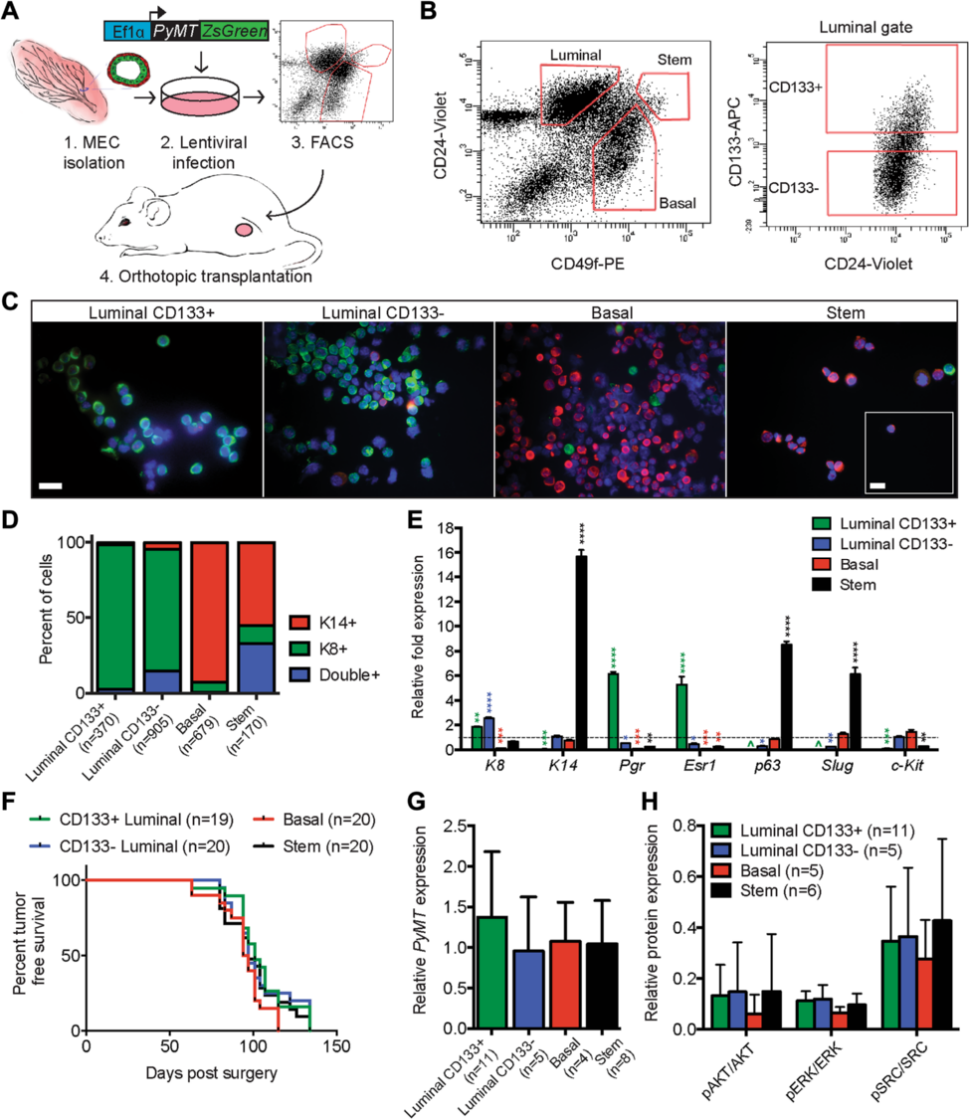
A model to assess the influence of the cell of origin on tumor phenotype. **a** Freshly isolated mammary epithelial cells (MECs) were transduced with the EF1α-*PyMT*-*ZsGreen* lentivirus, sorted into distinct populations by fluorescence-activated cell sorting (FACS), and transplanted into the cleared mammary fat pads of syngeneic mice. **b** Transduced MECs were sorted into basal, luminal, and stem cell populations based on the expression of the cell surface markers CD49f and CD24 (*right*). Luminal cells were further sorted according to CD133 expression into hormone receptor–positive (CD133+) and hormone receptor–negative (CD133−) populations (*left*). The collected populations are indicated by *red gates. APC* allophycocyanin, *PE* phycoerythrin. **c** FACS-enriched populations were evaluated for expression of basal keratin 14 (K14; *red*), luminal keratin 8 (K8; *green*), and 4′,6-diamidino-2-phenylindole dihydrochloride (*blue*) by immunofluorescence (scale bar = 20 μm). *Inset* shows representative K14/K8 double-positive cells from the stem cell enriched population (scale bar = 10 μm). **d** Quantification of the cytokeratin profile for each MEC subgroup (n = total number of cells imaged). **e** Real-time quantitative PCR (RT-qPCR) quantification of relative differences in cytokeratin (*K8, K14*), hormone receptor (*p* *rogesterone [Pgr], estrogen receptor [Esr1]*), transcription factor (*p63, Slug*), and tyrosine-protein kinase (*c-Kit*) RNA expression levels between sorted MEC populations normalized to the *GAPDH* housekeeping gene. Expression levels were compared (by *t* test) with RNA from unsorted controls (*dashed line*). *p < 0.05, **p < 0.01, ***p < 0.0005, ****p < 0.0001; ^expression values for *p63* and *Slug* in the luminal CD133+ population could not be determined, as amplification only rarely occurred below 40 cycles. Each data point is a mean ± SEM for triplicate qPCR reactions from three independent cDNA synthesis/preamplification reactions (n = 9). **f** Kaplan-Meier curves of mice receiving orthotopic transplants of distinct MEC subgroups. Mice were killed when tumors reached 2 cm in diameter (n = number of mice). **g** RT-qPCR quantification of relative *PyMT* mRNA expression levels in averaged luminal CD133+ cell, luminal CD133− cell, basal cell, and stem cell tumors normalized to the *Rplp0* housekeeping gene. No significant differences between tumor groups were detected (by *t* test, n = number of tumors). **h** Average ratio of phosphorylated (pAKT) to AKT, phosphorylated extracellular signal-regulated kinase (pERK) to ERK, and phosphorylated SRC (pSRC) to Src protein expression in luminal CD133+, luminal CD133−, basal, and stem cell tumors normalized to the β-actin. No significant differences were detected between tumor groups (by analysis of variance for multiple comparisons)

The degree of tumor heterogeneity in *PyMT*-based mouse models is associated largely with stage of progression [24], mouse strain [40–42], and the approach used to target the oncogene to mammary epithelium [12, 27, 30, 31]. To extend upon these studies, we asked whether *PyMT* oncogenesis within specific luminal, basal, and stem cell populations would affect tumor latency, pathology, metastasis, and molecular subgroup. The experimental design for this analysis was to transduce primary MECs overnight with the EF1α-*PyMT-ZsGreen *lentivirus [12], followed by FACS enrichment of specific cell populations and transplantation with an excess of uninfected and unsorted MECs (Fig. 1a). This technique was designed to ensure that primary cells would be exposed to similar conditions during ex vivo procedures and to provide comparable cellular environments for tumor development.

In order to determine whether expression of the lentiviral transgenes would affect the cell sorting procedure, we assessed ZsGreen fluorescence and PyMT protein levels in MECs for 5 days following transduction with the EF1α-*PyMT-ZsGreen *lentivirus (Additional file 1, panel a). Only 2 % of cells were ZsGreen-positive at 24 h after transduction, whereas 30 % were positive at day 5. In addition, PyMT protein expression was nearly undetectable until day 3 (Additional file 1, panels b and c). These data suggest that the lentiviral genes would not affect cell sorting within 24 h after transduction.

Freshly isolated primary MECs were infected with the EF1α-PyMT-ZsGreen lentivirus overnight in suspension [12] and then stained and sorted for the cell surface proteins CD24, CD49f, and CD133, which are markers known to delineate luminal, basal, and stem cell populations [34, 35]. Infected cells were not gated on ZsGreen, owing to the low level of expression at 24 h posttransduction. In accordance with published studies, luminal, basal, and stem cells were isolated based on their expression of CD49f and CD24 (Fig. 1b) [32, 33, 35]. The luminal cell population was further separated into hormone receptor–positive and hormone receptor–negative fractions by expression of CD133 (hereinafter called *luminal CD133+* and *luminal CD133−*, respectively) (Fig. 1b) [34]. We next verified that enriched populations contained the expected keratin markers by evaluating each population for the expression of luminal cell-specific K8 and basal cell-specific K14 by immunofluorescence (Fig. 1c) [43]. As expected, the representative population markers were enriched in the appropriate groups (Fig. 1d) [32–35]. Interestingly, approximately 15 % of luminal CD133− cells and 33 % of enriched stem cells were positive for both K8 and K14, suggesting the presence of a bipotent progenitor population in these groups [44–47]. In addition, expression levels of *K8*, *K14*, *Esr1*, the *progesterone receptor* (*Pgr*), transcription factors (*p63* and *Slug*), and tyrosine-protein kinase (*c-Kit*) RNA were measured in each population by RT-qPCR (Fig. 1e). The gene expression patterns we observed confirmed the enriched expression of hormone receptors *Esr1* and *Pgr* within the luminal CD133+ population [34], whereas the stem cell markers *p63* and *Slug* were highly expressed in the enriched stem cell population [9, 48]. Although both basal and stem cell populations expressed K14 protein (Fig. 1c), the mRNA was expressed in basal cells at a level similar to that of unsorted MECs, suggesting a difference in the transcriptional regulation of this gene between basal and stem cell populations.

To assess tumor formation and progression from each subpopulation, transduced luminal CD133+, luminal CD133−, basal, and stem cells were individually transplanted into cleared mouse mammary fat pads. Each enriched MEC population was cotransplanted with unsorted and untransduced MECs to provide a similar in vivo environment for outgrowth and to minimize the potential for transdifferentiation effects [49]. Tumors arose from each enriched MEC population, and no statistical difference in average tumor latency or tumor-free survival was observed (Fig. 1f), demonstrating that all enriched MEC populations have the capacity to undergo transformation and generate tumors. Also, no difference in tumor latency was observed when transduced MECs were transplanted into ovariectomized mice, demonstrating that each cell population can establish tumors independent of systemic hormones (Additional file 2, panel b). Moreover, the short tumor latency suggests that transformation is rapid and may be independent of acquired mutations, which is also consistent with the observation that three-dimensionally cultured MECs infected with a *PyMT*-expressing lentivirus exhibit a transformed phenotype within 2 weeks [12].

Expression of *PyMT* mRNA and AKT, SRC, and ERK proteins was analyzed in 31 tumors to assess the variability of the PyMT signaling pathway between tumors. Similar to previous findings [12], the expression of *PyMT* mRNA was variable in tumor samples (Additional file 3, panel a). However, the average expression of *PyMT* in tumors was not significantly different between tumors arising from the four cellular origins (Fig. 1g) [12]. Furthermore, protein analysis demonstrated that AKT, ERK, and SRC activation was not significantly different between tumors (Fig. 1h and Additional file 3, panels b–d). These data suggest that *PyMT* expression and signaling are similar in tumors arising from different MEC populations.

### Enriched mammary epithelial cell populations establish tumors with broad histopathology

**Fig. 2:**
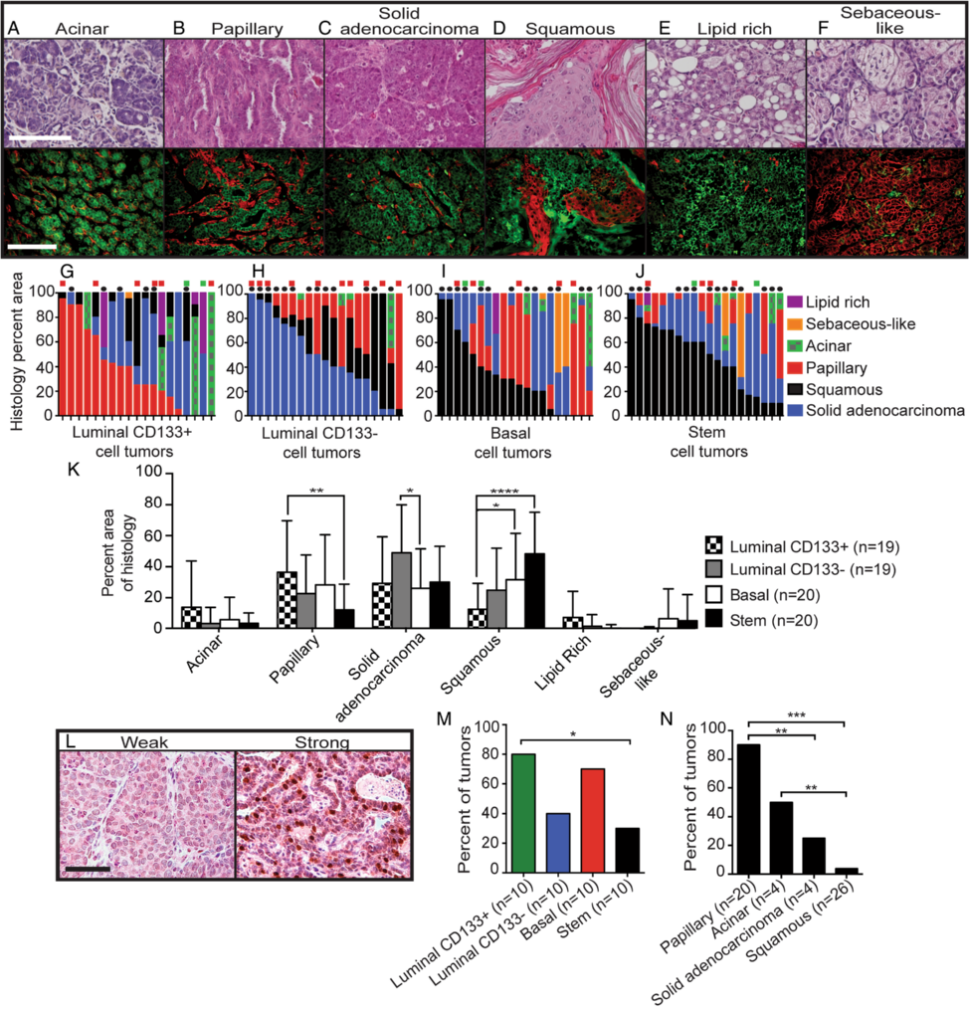
Analysis and prevalence of histology in tumors derived from mammary epithelial cell (MEC) populations. **a** through **f** Representative images of hematoxylin and eosin and cytokeratin staining of tumor histologies: acinar (**a**), papillary (**b**), solid adenocarcinoma (**c**), squamous (**d**), lipid rich (**e**), and sebaceous-like (**f**). Immunofluorescence staining was performed for basal keratin 14 (K14; *red*) and luminal keratin 8 (K8; *green*) (scale bar = 100 μm). ZsGreen fluorescence was not detected in the processed sections. Histological area per tumor was derived from luminal CD133+ cells (**g**), luminal CD133− cells (**h**), basal cells (**i**), and stem cells (**j**). *Boxes* above each column indicate tumors that were used for microarray analysis. *Red boxes* = basal subgroup; *green boxes* = luminal subgroup. *Black circles* mark tumors that were metastatic. **k** Average area of histology per MEC group (unpaired *t* test; n = number of tumors). **l** Representative images of estrogen receptor (ESR1) staining, including negative (*left panel*) and positive staining (*right panel*) (scale bar = 50 μm; n = number of tumors). **m** Quantification of ESR1 staining per MEC group (two proportion *z* test). **n** Quantification of ESR1 staining per histological specimen (two proportion *z* test). *p < 0.05, **p < 0.01, ***p < 0.0005, ****p < 0.0001

We next analyzed the histology of tumors originating from each of the enriched MEC populations. We classified tumors by H&E staining and cytokeratin expression and identified the following histologies: acinar, papillary, solid adenocarcinoma, squamous, lipid-rich, and sebaceous-like (Fig. 2a–f, Table 1). The frequency of a specific histology was quantified by estimating its area in two or three different sections per tumor (Fig. 2g–k). Data are presented as the total percentage area of a histological feature in each tumor (Fig. 2g–k) and as the predominant tumor histology of each tumor (Additional file 4). Tumors that produced lung metastases and those used for microarray analysis are also indicated.

**Table 1:** Description and summary of tumor histologies

Tumor histology	Overall cellular organization	Histology description	Cytokeratin staining
Acinar	Well differentiated	Acini with bilayered, duct-like epithelial cells	Maintenance of ductal morphology with K8 cells surrounding clear lumens and K14 cells adjacent to the stroma
Papillary	Well differentiated	Epithelial cell sheets, generally 2–3 layers thick, surrounded by stroma	Maintenance of luminal and basal cell organization with K14-positive cells adjacent to the stroma
Solid adenocarcinoma	Poorly differentiated	Multilayered epithelial cells grouped in large units (>100 μm in diameter) surrounded by 1–2 layers of stromal cells	Primarily K8-positive with sparse K14-positive cells near stromal regions
Squamous	Poorly differentiated	Adenocarcinoma with clusters of keratin pearls and sheets of squamous epithelia	Large solid areas of either K8- or K14-positive cells; K14 cells are localized near keratinized regions
Lipid-rich	Poorly differentiated	Heavily vacuolated epithelial cells	Primarily K8-positive
Sebaceous-like	Poorly differentiated	Epithelial cells with enlarged nuclei and foamy cytoplasms	Primarily K14-positive

*Abbreviations: K8* keratin 8, *K14* keratin 14

Several trends became apparent. First, histologies were not associated with ERK, AKT, or SRC phosphorylation levels, suggesting that histopathology is uncoupled from PyMT activity (Additional file 5, panel a). Second, although papillary features were observed in tumors derived from all cell types, they were significantly more prevalent in tumors arising from luminal CD133+ cells than in the stem cell–enriched population (Fig. 2k). Next, the predominant histology generated by luminal CD133− cells was solid adenocarcinoma, whereas basal and stem cell–enriched populations established more squamous tumors (Fig. 2k and Additional file 4). Finally, several rare tumor types originated from specific MEC populations. Lipid-rich tumors arose primarily from the luminal CD133+ cell population, whereas sebaceous-like carcinomas developed primarily from basal and stem cells (Fig. 2k and Additional file 4). Thus, each MEC-enriched population generated tumors with a broad but distinct spectrum of histological subtypes.

ESR1 is a standard clinical marker used to guide a patient’s course of treatment and predict clinical outcome [50]. ESR1 IHC staining was used to determine if any enriched MEC subgroups developed tumors with extensive ESR1-positive (ER+) cells, as defined by strong nuclear staining (Fig. 2l) in 20 % or more of tumor cells. Surprisingly, all of the enriched MEC populations gave rise to ER+ tumors, and luminal CD133+ cells generated the highest proportion of ER+ tumors (Fig. 2m). Most ER+ tumors exhibited either papillary or acinar histology, whereas few squamous tumors stained positive for ESR1 (Fig. 2n). These observations are consistent with previous reports that ER+ status appears to be associated with well-differentiated tumor histologies in *PyMT* oncogene-driven tumors and is less prevalent in tumors that are poorly differentiated [24]. Although ER+ tumors were observed, their hormone dependence was not apparent. Cells isolated from both ER+ and ER− tumors exhibited similar in vitro sensitivity to tamoxifen, and no difference in tumor latency was observed when transduced MECs were transplanted into ovariectomized mice (Additional file 2). Similar to these results, both the expression of *Esr1* in tumor cells and lack of estrogen-dependent growth were observed when *PyMT* was targeted to mammary glands through intraductal injection of a modified avian retroviral vector [31]. Thus, *Esr1* expression and estrogen-dependent growth appear uncoupled in virus-based* PyMT *tumor models.

### Luminal CD133− mammary epithelial cells give rise to an exclusively basal subgroup mammary tumors

**Fig. 3:**
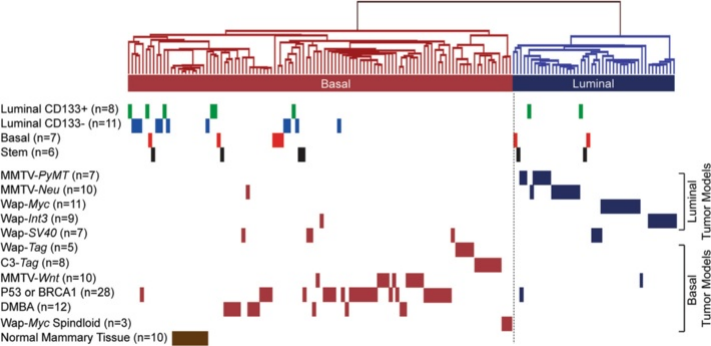
Tumor microarray gene expression profiling. Tumors were analyzed by microarray gene expression profiling and hierarchically clustered with mouse mammary tumor models using an intrinsic gene set identified by Herschkowitz et al. [7]. *Vertical lines* indicate individual tumors. Each enriched mammary epithelial cell population is indicated by a different color: *green* = luminal CD133+; *blue* = luminal CD133−; *red* = basal; *black* = stem cells. Mouse mammary tumor models that generate molecularly luminal tumors are shown in *dark blue*, and those predominantly within the basal subgroup are displayed in *dark red*. Normal mouse mammary tissue is represented in *brown* (n = number of tumors). *DMBA* 7,12-dimethylbenz[a]anthracene, *MMTV* mouse mammary tumor virus, *PyMT* polyomavirus middle T antigen

We next tested whether MEC populations influenced the molecular classification of the tumors they generate. Tumors derived from each MEC population were classified by hierarchical analysis of microarray gene expression data. This classification method has previously been used to cluster tumors from a variety of mouse models into basal and luminal subgroups [7]. We analyzed between 6 and 11 tumors generated from each enriched MEC population. An intrinsic 669-gene set consisting of genes differentially expressed in mouse tumors representing basal and luminal subgroups was used to determine the molecular classification [7], and gene set expression data were hierarchically clustered with 12 mouse models of breast cancer. On the basis of this analysis, we found that luminal CD133+, basal cell, and stem cell enriched MECs were able to give rise to tumors of both basal and luminal subgroups (Fig. 3), with no correlation to tumor histology or ESR1 status (data not shown). However, protein analysis of AKT, SRC, and ERK did reveal a difference in AKT phosphorylation in the basal subgroup (Additional file 5, panel b). Interestingly, the luminal CD133− population, which is enriched in luminal progenitors [34], exclusively established tumors within the basal subgroup. To determine the significance of this observation, we compared the distribution of basal and luminal subgroups generated from each cell population with the distribution we observed when unsorted MECs were transduced with the EF1α-*PyMT-ZsGreen* lentivirus [8]. Compared with unsorted MECs, the basal-restricted distribution of tumors derived from luminal CD133− cells was statistically significant (*p* = 0.017) (Additional file 6: Table S1). In contrast, all other cell populations generated tumors of both basal and luminal subgroups at a frequency similar to that of unsorted MECs. These data demonstrate that the stem, basal, and luminal CD133+ MEC populations can establish tumors that classify within either molecular subgroup, whereas CD133− luminal cells preferentially establish tumors of the basal subgroup.

Unsupervised hierarchical clustering of tumors generated from enriched MEC populations revealed two major hierarchical tumor clusters, one composed of only CD133− luminal tumors and another composed of tumors generated from all MEC populations (Additional file 7). Differential expression analysis comparing these two major hierarchical clusters identified 1046 unique genes showing at least twofold differential expression at an adjusted *p* value <0.05 (Additional file 8: Table S2 and Additional file 9: Table S3). One gene of interest, *Sox11*, was upregulated in tumors derived from luminal CD133− MECs. Interestingly, *Sox11* is a transcriptional regulator upregulated in *BRCA1*-mutant breast cancers and has been shown to promote cell survival and proliferation in breast cancer cells [51].

### Luminal CD133+ cells give rise to tumors that are significantly less metastatic than other tumor groups

**Fig. 4:**
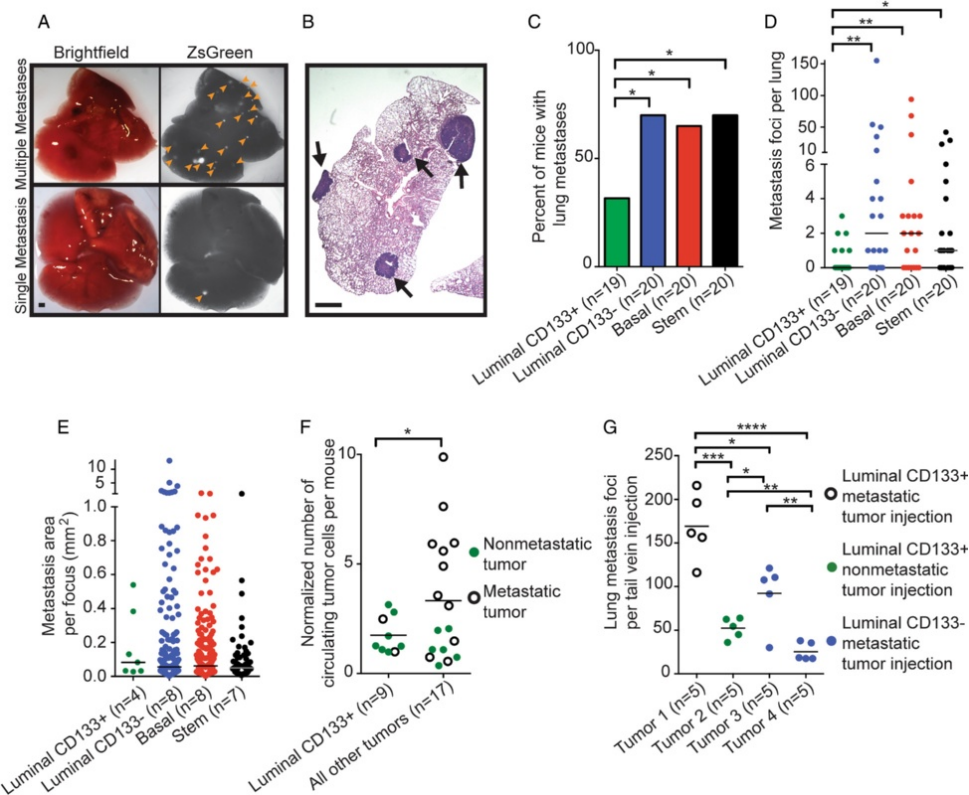
Metastatic profiles of tumors generated from enriched mammary epithelial cell populations. **a** Representative bright-field (*left panel*) and fluorescent (*right panel*) images of the same lung. *Arrowheads* indicate metastases (scale bar = 1 mm). **b** Representative image of hematoxylin and eosin staining of a metastatic lung section. *Arrows* indicate metastases (scale bar = 1 mm). **c** Percentage of mice with lung metastases per tumor group. Luminal CD133+ cell tumors were less metastatic than the other tumor groups (two proportion *z* test; n = number of mice). **d** Number of metastatic lung foci per tumor group. Luminal CD133+ cell tumors generated fewer metastatic foci than the other tumor groups (Mann–Whitney *U* test; median values shown). **e** Quantification of metastasis area per unique metastatic site in serial lung sections. No difference in size of tumor metastasis was detected between the tumor groups (n = number of mice). **f** Normalized number of circulating tumor cells in mice with luminal CD133+ tumors compared with all other tumor groups. ZsGreen signaling in whole blood isolated from tumor bearing mice was analyzed by fluorescence-activated cell sorting and normalized to no-tumor control signal. Luminal CD133+ tumor-bearing mice had fewer circulating tumor cells. Mice with non-metastatic tumors are represented by *green*, and those with metastatic tumors are represented by *black* (unpaired *t* test; mean values shown; n = number of mice). **g** Quantification of the number of lung tumor foci per tail vein injection of metastatic luminal CD133+ tumor cells (tumor 1), non-metastatic luminal CD133+ tumor cells (tumor 2), or metastatic luminal CD133− tumor cells (tumors 3 and 4) (unpaired *t* test; mean values shown; n = number of mice). Data shown in (**a**)–(**e**) represent spontaneous metastasis occurring from primary tumors, and data shown in (**f**) are from non-spontaneous tail vein injection assays. *p < 0.05, **p < 0.01, ***p < 0.0005, ****p < 0.0001

We investigated whether the enriched MEC populations expressing *PyMT* could generate tumors with different metastatic capacities. Metastasis was measured in mice with similar tumor latency, tumor size (2 cm), and tumor burden (single tumor). Similar to other *PyMT*-driven models, primary tumors generated from each MEC population metastasized to the lungs (Fig. 4a, b) [16]. Metastases to other organs were not detected. The metastatic burden in lungs was quantified by whole mount fluorescent imaging and H&E staining of serial lung sections from mice with similar tumor size and latency. Interestingly, although no significant differences in tumor prevalence or progression were observed between the four tumor-initiating populations (Fig. 1d), tumors derived from the luminal CD133+ cell population were significantly less metastatic than all other tumor groups (Fig. 4c). Furthermore, these cells formed significantly fewer lung tumor foci than the other tumor groups (Fig. 4d); however, there was no difference in the average area per metastatic focus (Fig. 4e).

To determine whether tumors with specific histologies have different metastatic propensities, we examined the numbers of metastases per tumor histology type (Additional file 10, panels a–d). We did not find an association between metastatic propensities and histology (Additional file 11). In addition, an association was not observed between PyMT downstream activity and number of metastases (Additional file 5, panel c). Taken together, these data suggest that metastatic properties of a tumor may be influenced by the tumor’s cellular precursor.

Metastasis progresses through several stages: Cells leave the primary site and enter the bloodstream or lymphatics, survive within the circulatory system, exit the circulatory system, and colonize a secondary site [52]. We next tested whether tumors derived from CD133+ cells were deficient in one or more steps of the metastatic process. First, we quantified CTCs in mice bearing primary tumors, which provides a measure of tumor cell invasion and intravasation into the bloodstream. CTCs expressing ZsGreen, a fluorescent protein coexpressed with *PyMT*, were quantified in whole blood from tumor-bearing mice by FACS and normalized to both the volume of collected blood and background fluorescence observed in non-tumor-bearing mice. Whole blood from mice with luminal CD133+ cell transplants had significantly fewer CTCs than the other tumor groups (Fig. 4f). Next, using a non-spontaneous metastasis assay, metastatic colony formation was assessed following tail vein injection of isolated tumor cells to evaluate their ability to extravasate from the bloodstream and colonize secondary sites. When injected into the tail vein, tumor cells derived from both luminal CD133+ and luminal CD133− populations were able to colonize and proliferate within the lung, regardless of whether the donor tumor was metastatic (Fig. 4g and Additional file 12). These data suggest that luminal CD133+ cells give rise to tumors with a limited ability to escape the primary cancer and intravasate into the bloodstream.

## Discussion

The *PyMT* oncogene has a broad capability to establish a variety of tumor histologies and subtypes. Members of the Li laboratory developed a model wherein a modified avian leukosis sarcoma virus expressing *PyMT* (RCAS-*PyMT*) was used to infect transgenic mice engineered to express *tva*, the RCAS (replication competent avian sarcoma leukosis virus LTR splice acceptor) receptor, on different mammary cell types [53]. Intraductal infection with RCAS-*PyMT* was performed in mice expressing *tva* under the control of several promoters, including the MMTV LTR and K6 promoter [27, 31]. When tumors generated by the different models were compared, dissimilar histological and molecular phenotypes were observed. MMTV-*tva*/RCAS-*PyMT* tumors were acinar and composed of luminal and myoepithelial cells, a phenotype that contrasted with the papillary tumors generated by K6-*tva*/RCAS-*PyMT*. These researchers also showed that the molecular profiles of tumors generated by MMTV-*tva*/RCAS-*PyMT*, K6-*tva*/RCAS-*PyMT*, and MMTV-*PyMT* were not similar, with the K6-*tva* tumors having a unique profile similar to that of a normal-like breast cancer subtype [27]. In an expanded analysis of molecular profiles from the RCAS-*PyMT* and MMTV-*PyMT* models, Hollern and Andrechek further demonstrated significant diversity, particularly among tumors derived from different mouse strains [40]. Taken together, these data demonstrate that the *PyMT* oncogene has the capacity to generate several tumor subtypes and that differences in the cellular context of the oncogene can affect tumor heterogeneity.

We have similarly observed a variety of histological and molecular subtypes in tumors induced by the* PyMT *oncogene. Previously, we showed that broad expression of the oncogene in mammary epithelium, using the EF1α-*PyMT-ZsGreen *lentivirus, generated late-stage tumors consisting of both luminal and myoepithelial cells, with a majority of tumors having acinar and solid histology [12], which are similar features of tumors established by the MMTV-*tva*/RCAS-*PyMT* model [53]. However, a notable difference between these models was the appearance of squamous metaplasia, which was observed in 30 % of the EF1α-*PyMT-ZsGreen* tumors, but not in MMTV-*tva*/RCAS-*PyMT* tumors [12, 27]. This tumor subtype is rare in the MMTV-*PyMT* model, with a reported frequency of only 4–8 % [22, 40]. The data in our present study expand upon those previous studies by demonstrating that squamous metaplasia was observed more frequently when basal and stem cell populations were infected with the EF1α-*PyMT-ZsGreen *lentivirus. Similar data were obtained by Keller et al. from studies performed with normal human breast cells. They showed that transduction of CD10-enriched human basal cells with oncogenic lentiviruses resulted in ER− and metaplastic tumors with squamous differentiation. In contrast, epithelial cell adhesion molecule–positive (Epcam+) luminal cells generated ductal carcinomas with luminal features [54]. In addition, Ince et al. showed that transformation of myoepithelial-like human breast cells generated squamous carcinomas of the breast, whereas luminal-like cells gave rise to adenocarcinomas [14]. Collectively, these studies provide strong support that a cell within the basal lineage, either a myoepithelial cell or a stem cell, is the origin of squamous metaplasia in mammary tumors.

The ability to fractionate primary mouse mammary cells into luminal, basal, and stem cell populations, and their receptiveness to ex vivo manipulation and outgrowth following transplantation, provides a unique method to study how cellular context affects oncogenesis [12]. In the present study, we investigated whether tumor latency, histology, metastasis, and molecular subtype are altered when the *PyMT* oncogene is targeted to distinct FACS-enriched mammary cell populations. The data demonstrate that each MEC population is able to generate tumors at similar latency and with broad pathology. However, some cell populations preferentially established distinct pathologies. Most striking was that luminal CD133+ cells gave rise to a higher proportion of tumors with papillary histology and ESR1 expression and the lowest proportion of tumors with squamous metaplasia. Consistent with their well-differentiated pathology, tumors from luminal CD133+ cells also produced fewer CTCs and metastases. An opposing phenotype was observed in tumors generated by enriched stem cells. Notably, these cells generated tumors that were ER−, exhibited squamous metaplasia, and produced more CTCs and metastases than luminal CD133+ cells. Thus, luminal CD133+ cells had a propensity to produce well-differentiated tumors, whereas poorly differentiated and metaplastic tumors were more commonly observed from enriched stem cells.

Although the MMTV LTR is expressed primarily in luminal cells, it is also active broadly in basal, stem, and luminal cell populations of the mammary gland [27, 29]. In the MMTV-*PyMT* model, the oncogene may target a multipotent progenitor because clonal cell lines derived from precancerous lesions can establish cell types expressing markers for both luminal and myoepithelial populations [25, 55]. Histological and cellular heterogeneity are also observed in vivo in the MMTV-*PyMT* model, particularly during precancerous development. Early lesions are composed of both hormone receptor–positive and hormone receptor–negative cells, with sporadic myoepithelial coverage [24]. As the tumors develop to malignant lesions, they exhibit a loss of both hormone receptor–expressing cells and myoepithelial cells [24] and an expansion of cells double-positive for CD61 and CD29 [26], markers that are coexpressed on normal luminal progenitors. Some similarities between MMTV-*PyMT* tumors and those derived from the luminal CD133− cell population are evident. In particular, the histopathology of tumors from this population was most similar to that of tumors in the MMTV-*PyMT* mouse model. Solid adenocarcinomas are seen in 60 % of MMTV-*PyMT* tumors [22], and this was the dominant histology in approximately 40 % of tumors derived from the luminal CD133− cell population. However, tumors established by luminal CD133− cells were classified as a basal subgroup, whereas MMTV-*PyMT* tumors clustered within the luminal subgroup [7]. The reason for this discrepancy is unclear, but it may be a result of differences in the developmental stage of cells targeted in each model. The MMTV LTR is active at prepubertal stages of mammary gland development [29], which suggests that immature mammary epithelium is the origin for MMTV-*PyMT* tumors. In contrast, the primary MECs used for FACS enrichment in our study were derived from postpubertal mice. These cells would have had been exposed to the maturation effects of systemic hormones, which may alter their lineage potential. Recent studies have shown that oncogenic stress can significantly increase the plasticity and multilineage potential of differentiated MECs [56–58]. Both the MMTV-*tva*/RCAS-*PyMT* and EF1α-*PyMT-ZsGreen* models generate heterogeneous tumors consisting of myoepithelial and luminal cell lineages, which contrasts with the predominantly luminal cell type that is observed in late-stage MMTV-*PyMT* tumors [59]. These data suggest that tumor cells generated by the MMTV-*PyMT* and virus-based models have differences in their lineage plasticity, particularly at more advanced stages of progression, which may influence the molecular classification of tumors. Further studies are necessary to assess the effect of oncogenic stress on the lineage plasticity of MECs at pre- and postpubertal stages of development.

Using the method described by Herschkowitz et al., we categorized tumors derived from the sorted cell populations and 12 different mouse models of breast cancer into 2 general molecular subgroups: basal and luminal [7, 60]. These data demonstrate that both luminal and basal tumor subgroups can arise from enriched luminal CD133+, stem, and basal cell populations. However, we also show that transformation of CD133− luminal cells, which are enriched for luminal progenitors [34], generated tumors of the basal subgroup. This finding suggests luminal progenitors preferentially establish basal rather than luminal tumors. Consistent with this, several recent observations attribute basal-like breast cancer to a luminal population. Lim et al. demonstrated that the molecular profile of untransformed luminal progenitors most closely resembles basal-like breast cancers [8]. In addition, transformation of human EpCAM+/CD10−/CD49f + luminal progenitors derived from reduction mammoplasties established tumors with features similar to basal-like breast cancer, including reduced ESR1 and greater K14 expression than tumors derived from differentiated luminal cells [54]. Furthermore, targeting *Brca1* loss of function to luminal cells in mice generated tumors with basal-like features that closely resembled those observed in patients carrying the *BRCA1* mutation. However, the same loss of function in basal cells generated adenomyoepitheliomas [10, 15]. Taken together, these data support luminal progenitors as a cellular origin of basal-like breast cancer.

There are a number of challenges to studying how the cell of origin influences tumor progression in breast cancer. These include the complexity of normal mammary cell types from which a tumor may arise [60] and technical difficulties in precisely targeting oncogenes to specific cell types. As such, the data from this study should be considered in the context of limitations of the approach used to target expression of *PyMT* to distinct cell populations. For example, the cells that were transplanted represent enriched populations but not purified cell types. Thus, a tumor phenotype cannot be credited to a specific cell type. In addition, specific cell populations may have a transduction bias, as has been observed between cultured luminal and myoepithelial cells, which may limit the diversity of cell types targeted by the lentivirus [61]. Transduction of primary MECs with lentiviruses and the subsequent expression of* PyMT *and *ZsGreen* may also alter the sorting process. However, maximum expression of lentiviral transgenes generally takes several days. ZsGreen expression was observed in only about 2 % of cells 24 h after transduction, and PyMT protein expression was low at this time. Because cell sorting and transplantation were performed within 24 h after the cells were exposed to virus, it is unlikely that expression of* PyMT *would have any significant influence on the populations before the sort. In addition, the study was designed to ensure each sorted population was exposed to similar conditions during the ex vivo procedures. Primary cells were collected, transduced, and sorted by FACS from a pool, and each population was cotransplanted with 5–20-fold more uninfected, unsorted MECs, which would provide comparable in vivo environments for tumor development. Thus, the main variable was the cell population used to generate the tumors.

## Conclusions

We report that differentiated luminal (CD133+), luminal progenitor (CD133−), basal, and stem cell enriched populations have the capacity to give rise to mammary tumors at equivalent frequency and latency when transformed with *PyMT* oncogene. However, mammary cell populations can produce tumors with differences in histopathology, molecular classification, and metastatic potential. Basal and stem cell enriched populations predominantly established poorly differentiated squamous tumors, whereas differentiated luminal cells gave rise to a high proportion of ER+ papillary tumors. We also demonstrate that the luminal progenitor enriched population specifically gives rise to tumors that cluster within the basal molecular subgroup. Taken together, these data demonstrate that targeted expression of *PyMT* in different cell types affects tumor histology, molecular subtype, and metastatic potential. In addition, the data provide support that the cellular origin of squamous metaplasia resides within the basal or mammary stem cell population.

## Additional files

Additional file 1: Figure S1. Postinfection *PyMT* expression in cultured total MECs. **a** Flow cytometric analysis of percentage of ZsGreen-positive cells for 5 days post-EF1α-*PyMT-ZsGreen* infection (10,000 events, mean ± SD, n = 3). **b** Quantification from simple Western blot analysis of PyMT-HA expression over time in infected MECs and uninfected MEC controls for 5 days following infection. PyMT-HA peak area was normalized to GAPDH peak area. 293 T cells are shown as positive and negative controls for PyMT-HA expression. **c** Multiplex simple Western blot stained for hemagglutinin (HA) tag and GAPDH. Protein lysates were prepared each day for 5 days following infection and PyMT-HA (60 kDa) with 293 T positive and negative controls. HA tag and GAPDH signals are shown with different exposure times. (TIFF 1824 kb) Additional file 2: Figure S2. Hormone independence of EF1α-*PyMT-ZsGreen*-transduced MEC tumors. **a** Half-maximal inhibitory concentration values of wild-type MECs and cells isolated from luminal CD133+, luminal CD133−, basal, and stem cell tumors treated for 48 h with 4-hydroxytamoxifen (4-OHT). No significant differences among the viability of tumor cells and normal MECs were observed after 4-OHT treatment (*t* test). **b** Assessment of tumor growth response to ovariectomy. Transduced primary cells were transplanted into ovariectomized mice to test if lack of hormone signaling would result in reduced tumor growth from luminal CD133+ cells. No differences in tumor latencies were observed among tumor groups (n = number of mice). (TIFF 1112 kb) Additional file 3: Figure S3.
*PyMT* expression and activity in tumors. **a** RT-qPCR quantification of relative *PyMT* mRNA expression levels in individual MMTV-PyMT, luminal CD133+ cell, luminal CD133− cell, basal cell, and stem cell tumors normalized to the *Rplp0* housekeeping gene. Analysis was performed in triplicate for each tumor sample. **b** Western blot quantification of relative ratio of pAKT to AKT protein expression in individual MMTV-*PyMT*, luminal CD133+ cell, luminal CD133− cell, basal cell, and stem cell tumors normalized to β-actin. **c** Western blot quantification of relative ratio of pERK to ERK protein expression in individual MMTV-*PyMT*, luminal CD133+ cell, luminal CD133− cell, basal cell, and stem cell tumors normalized to β-actin. **d** Western blot quantification of relative ratio of pSRC to SRC protein expression in individual MMTV-*PyMT*, luminal CD133+ cell, luminal CD133− cell, basal cell, and stem cell tumors normalized to β-actin. (TIFF 2407 kb) Additional file 4: Figure S4. Percentage of tumors with a dominant histology. Each tumor was classified based on the histological feature representing at least 50 % of the tumor area. Tumors classified as mixed did not have a dominant histological type. Tumors derived from stem cells were significantly more squamous than tumors derived from luminal CD133+ and CD133− luminal cells (two proportion *z* test, n = number of tumors). (TIFF 833 kb) Additional file 5: Figure S5. PyMT activity comparison with tumor histology, molecular subtype, and metastasis. **a** Western blot quantification of relative ratio of pAKT to AKT, pERK to ERK, and pSRC to SRC protein expression in enriched population tumors segregated based on tumor histology and normalized to β-actin. No significant differences in protein expression were detected among tumor groups (*t* test, n = number of tumors). **b** Western blot quantification of relative ratio of pAKT to AKT, pERK to ERK, and pSRC to SRC protein expression in enriched population tumors segregated based on tumor molecular subtype and normalized to β-actin. Molecularly basal tumors exhibited significantly higher levels of pERK/ERK levels than luminal tumors (*t* test, *p* value shown, n = number of tumors). **c** Western blot quantification of relative ratio of pAKT to AKT, pERK to ERK, and pSRC to SRC protein expression in enriched population tumors segregated based on tumor metastasis and normalized to β-actin. No significant differences in protein expression were detected among tumor groups (*t* test, n = number of tumors). (TIFF 1046 kb) Additional file 6: Table S1. Distribution of basal and luminal subtypes. Distribution of basal and luminal tumor subtypes generated by EF1α-*PyMT-ZsGreen* lentiviral transduction of each sorted cell population as compared with the distribution obtained from EF1α-*PyMT-ZsGreen*-transduced but unsorted MECs [8], using Fisher’s exact test. (DOCX 14 kb) Additional file 7: Figure S6. Unsupervised hierarchical clustering of tumors generated from enriched MEC populations. Tumor cell of origin, histology, metastatic propensity, and molecular subtype are indicated below the dendrogram cluster. (TIFF 1486 kb) Additional file 8: Table S2. Top 100 unique differentially expressed genes in the luminal CD133− tumor cluster compared with the mixed tumor cluster. Group 1 is the mixed tumor cluster, and group 2 is the luminal CD133− tumor cluster. A *t* test was performed on Benjamini–Hochberg-corrected, log-transformed data. Table includes gene name, ratio, *p* value, and direction. (XLSX 14 kb) Additional file 9: Table S3. All differentially expressed genes in the luminal CD133− tumor cluster compared with the mixed tumor cluster. Group 1 is the mixed tumor cluster, group 2 is the luminal CD133− tumor cluster. A *t* test was performed on Benjamini–Hochberg-corrected, log-transformed data. Table includes gene name, ratio, *p* value, and direction. (XLSX 73 kb) Additional file 10: Figure S8. Number of metastases per tumor histology. Histological area per tumor derived from luminal CD133+ cells (**a**), luminal CD133− cells (**b**), basal cells (**c**), and stem cells (**d**), as well as the number of metastatic foci detected in the lungs of the mice bearing each of the tumors (shown as red values above each tumor). (TIFF 1562 kb) Additional file 11: Figure S9. Quantification of the number of metastases per tumor histology. A histology was assigned to a tumor when the histology comprised at least 50 % of the tumor area. Tumors that had mixed histologies were classified as mixed. No statistically significant differences in the metastatic propensities among histological tumor types were observed (Mann–Whitney *U* test). (TIFF 654 kb) Additional file 12: Figure S7. Enriched MEC populations metastasize to lung. Representative fluorescence images of lungs from mice that received tail vein injections of metastatic luminal CD133+ tumor cells (tumor 1), nonmetastatic luminal CD133+ tumor cells (tumor 2), or metastatic luminal CD133− tumor cells (tumors 3 and 4). Lungs were examined 3 weeks after the tail vein injection. Fluorescent black-and-white lung images were false-colored green in ImageJ software and overlaid on a black background. (TIFF 2284 kb)

## Abbreviations

7-AAD: 7-Amino-actinomycin D
APC: Allophycocyanin
BSA: Bovine serum albumin
CTC: Circulating tumor cell
DMBA: 7,12-Dimethylbenz[a]anthracene
EDTA: Ethylenediaminetetraacetic acid
EF1α: Elongation factor 1 alpha
EpCAM: Epithelial cell adhesion molecule
ER and Esr1: Estrogen receptor
ER−: Estrogen receptor–negative
ER+: Estrogen receptor–positive
ERK1/2: Extracellular signal-regulated kinase 1/2
FACS: Fluorescence-activated cell sorting
GAPDH: Glyceraldehyde 3-phosphate dehydrogenase
GEO: Gene Expression Omnibus
HA: Hemagglutinin
H&E: Hematoxylin and eosin
HBSS: Hanks’ balanced salt solution
HCI: Huntsman Cancer Institute
IgG: Immunoglobulin G
IHC: Immunohistochemistry
K8: Keratin 8
K14: Keratin 14
LTR: Long terminal repeat
MEC: Mammary epithelial cell
MMTV: Mouse mammary tumor virus
PBS: Phosphate-buffered saline
PR and Pgr: Progesterone receptor
PyMT: Polyomavirus middle T antigen
RCAS: Replication competent avian sarcoma leukosis virus long terminal repeat splice acceptor
RT-qPCR: Real-time quantitative PCR

## References

[1] Sørlie T, Perou CM, Tibshirani R, Aas T, Geisler S, Johnsen H (2001). Gene expression patterns of breast carcinomas distinguish tumor subclasses with clinical implications. Proc Natl Acad Sci U S A.

[2] Malhotra GK, Zhao X, Band H, Band V (2010). Histological, molecular and functional subtypes of breast cancers. Cancer Biol Ther.

[3] Weigelt B, Reis-Filho JS (2009). Histological and molecular types of breast cancer: is there a unifying taxonomy?. Nat Rev Clin Oncol.

[4] Santagata S, Ince TA (2014). Normal cell phenotypes of breast epithelial cells provide the foundation of a breast cancer taxonomy. Expert Rev Anticancer Ther.

[5] Visvader JE, Stingl J (2014). Mammary stem cells and the differentiation hierarchy: current status and perspectives. Genes Dev.

[6] Ignatiadis M, Sotiriou C (2013). Luminal breast cancer: from biology to treatment. Nat Rev Clin Oncol.

[7] Herschkowitz JI, Simin K, Weigman VJ, Mikaelian I, Usary J, Hu Z (2007). Identification of conserved gene expression features between murine mammary carcinoma models and human breast tumors. Genome Biol.

[8] Lim E, Vaillant F, Wu D, Forrest NC, Pal B, Hart AH (2009). Aberrant luminal progenitors as the candidate target population for basal tumor development in *BRCA1* mutation carriers. Nat Med.

[9] Lim E, Wu D, Pal B, Bouras T, Asselin-Labat ML, Vaillant F (2010). Transcriptome analyses of mouse and human mammary cell subpopulations reveal multiple conserved genes and pathways. Breast Cancer Res.

[10] Hennessy BT, Gonzalez-Angulo AM, Stemke-Hale K, Gilcrease MZ, Krishnamurthy S, Lee JS (2009). Characterization of a naturally occurring breast cancer subset enriched in epithelial-to-mesenchymal transition and stem cell characteristics. Cancer Res.

[11] Shi P, Wang M, Zhang Q, Sun J (2008). Lipid-rich carcinoma of the breast: a clinicopathological study of 49 cases. Tumori.

[12] Smith BA, Shelton DN, Kieffer C, Milash B, Usary J, Perou CM (2012). Targeting the PyMT oncogene to diverse mammary cell populations enhances tumor heterogeneity and generates rare breast cancer subtypes. Genes Cancer.

[13] The Cancer Genome Atlas Network (2012). Comprehensive molecular portraits of human breast tumours. Nature.

[14] Ince TA, Richardson AL, Bell GW, Saitoh M, Godar S, Karnoub AE (2007). Transformation of different human breast epithelial cell types leads to distinct tumor phenotypes. Cancer Cell.

[15] Molyneux G, Geyer FC, Magnay FA, McCarthy A, Kendrick H, Natrajan R (2010). *BRCA1* basal-like breast cancers originate from luminal epithelial progenitors and not from basal stem cells. Cell Stem Cell.

[16] Guy CT, Cardiff RD, Muller WJ (1992). Induction of mammary tumors by expression of polyomavirus middle T oncogene: a transgenic mouse model for metastatic disease. Mol Cell Biol.

[17] Fluck MM, Schaffhausen BS (2009). Lessons in signaling and tumorigenesis from polyomavirus middle T antigen. Microbiol Mol Biol Rev.

[18] Raptis L, Marcellus R, Corbley MJ, Krook A, Whitfield J, Anderson SK (1991). Cellular *ras* gene activity is required for full neoplastic transformation by polyomavirus. J Virol.

[19] Webster MA, Hutchinson JN, Rauh MJ, Muthuswamy SK, Anton M, Tortorice CG (1998). Requirement for both Shc and phosphatidylinositol 3′ kinase signaling pathways in polyomavirus middle T-mediated mammary tumorigenesis. Mol Cell Biol.

[20] Wood LD, Parsons DW, Jones S, Lin J, Sjöblom T, Leary RJ (2007). The genomic landscapes of human breast and colorectal cancers. Science.

[21] Fromowitz FB, Viola MV, Chao S, Oravez S, Mishriki Y, Finkel G (1987). ras p21 expression in the progression of breast cancer. Hum Pathol.

[22] Rosner A, Miyoshi K, Landesman-Bollag E, Xu X, Seldin DC, Moser AR (2002). Pathway pathology: histological differences between ErbB/Ras and Wnt pathway transgenic mammary tumors. Am J Pathol.

[23] Desai KV, Xiao N, Wang W, Gangi L, Greene J, Powell JI (2002). Initiating oncogenic event determines gene-expression patterns of human breast cancer models. Proc Natl Acad Sci U S A.

[24] Lin EY, Jones JG, Li P, Zhu L, Whitney KD, Muller WJ (2003). Progression to malignancy in the polyoma middle T oncoprotein mouse breast cancer model provides a reliable model for human diseases. Am J Pathol.

[25] Maglione JE, McGoldrick ET, Young LJ, Namba R, Gregg JP, Liu L (2004). Polyomavirus middle T-induced mammary intraepithelial neoplasia outgrowths: single origin, divergent evolution, and multiple outcomes. Mol Cancer Ther.

[26] Kouros-Mehr H, Bechis SK, Slorach EM, Littlepage LE, Egeblad M, Ewald AJ (2008). GATA-3 links tumor differentiation and dissemination in a luminal breast cancer model. Cancer Cell.

[27] Bu W, Chen J, Morrison GD, Huang S, Creighton CJ, Huang J (2011). Keratin 6a marks mammary bipotential progenitor cells that can give rise to a unique tumor model resembling human normal-like breast cancer. Oncogene.

[28] Gunther EJ, Belka GK, Wertheim GB, Wang J, Hartman JL, Boxer RB (2002). A novel doxycycline-inducible system for the transgenic analysis of mammary gland biology. FASEB J.

[29] Wagner KU, McAllister K, Ward T, Davis B, Wiseman R, Hennighausen L (2001). Spatial and temporal expression of the Cre gene under the control of the MMTV-LTR in different lines of transgenic mice. Transgenic Res.

[30] Siwko SK, Bu W, Gutierrez C, Lewis B, Jechlinger M, Schaffhausen B (2008). Lentivirus-mediated oncogene introduction into mammary cells in vivo induces tumors. Neoplasia.

[31] Toneff MJ, Du Z, Dong J, Huang J, Sinai P, Forman J (2010). Somatic expression of PyMT or activated ErbB2 induces estrogen-independent mammary tumorigenesis. Neoplasia.

[32] Shackleton M, Vaillant F, Simpson KJ, Stingl J, Smyth GK, Asselin-Labat ML (2006). Generation of a functional mammary gland from a single stem cell. Nature.

[33] Sleeman KE, Kendrick H, Ashworth A, Isacke CM, Smalley MJ (2006). CD24 staining of mouse mammary gland cells defines luminal epithelial, myoepithelial/basal and non-epithelial cells. Breast Cancer Res.

[34] Sleeman KE, Kendrick H, Robertson D, Isacke CM, Ashworth A, Smalley MJ (2007). Dissociation of estrogen receptor expression and in vivo stem cell activity in the mammary gland. J Cell Biol.

[35] Stingl J, Eirew P, Ricketson I, Shackleton M, Vaillant F, Choi D (2006). Purification and unique properties of mammary epithelial stem cells. Nature.

[36] Idris AI (2012). Ovariectomy/orchidectomy in rodents. Methods Mol Biol.

[37] Johnson WE, Li C, Rabinovic A (2007). Adjusting batch effects in microarray expression data using empirical Bayes methods. Biostatistics.

[38] Eyob H, Ekiz HA, Derose YS, Waltz SE, Williams MA, Welm AL (2013). Inhibition of Ron kinase blocks conversion of micrometastases to overt metastases by boosting antitumor immunity. Cancer Discov.

[39] Livak KJ, Schmittgen TD (2001). Analysis of relative gene expression data using real-time quantitative PCR and the 2^−ΔΔ*C*T^ method. Methods.

[40] Hollern DP, Andrechek ER (2014). A genomic analysis of mouse models of breast cancer reveals molecular features of mouse models and relationships to human breast cancer. Breast Cancer Res.

[41] Lifsted T, Le Voyer T, Williams M, Muller W, Klein-Szanto A, Buetow KH (1998). Identification of inbred mouse strains harboring genetic modifiers of mammary tumor age of onset and metastatic progression. Int J Cancer.

[42] Qiu TH, Chandramouli GV, Hunter KW, Alkharouf NW, Green JE, Liu ET (2004). Global expression profiling identifies signatures of tumor virulence in MMTV-PyMT-transgenic mice: correlation to human disease. Cancer Res.

[43] Mikaelian I, Hovick M, Silva KA, Burzenski LM, Shultz LD, Ackert-Bicknell CL (2006). Expression of terminal differentiation proteins defines stages of mouse mammary gland development. Vet Pathol.

[44] Li Z, Tognon CE, Godinho FJ, Yasaitis L, Hock H, Herschkowitz JI (2007). *ETV6-NTRK3* fusion oncogene initiates breast cancer from committed mammary progenitors via activation of AP1 complex. Cancer Cell.

[45] Sun P, Yuan Y, Li A, Li B, Dai X (2010). Cytokeratin expression during mouse embryonic and early postnatal mammary gland development. Histochem Cell Biol.

[46] Chakrabarti R, Wei Y, Romano RA, DeCoste C, Kang Y, Sinha S (2012). Elf5 regulates mammary gland stem/progenitor cell fate by influencing notch signaling. Stem Cells.

[47] Spike BT, Kelber JA, Booker E, Kalathur M, Rodewald R, Lipianskaya J (2014). CRIPTO/GRP78 signaling maintains fetal and adult mammary stem cells ex vivo. Stem Cell Reports.

[48] Guo W, Keckesova Z, Donaher JL, Shibue T, Tischler V, Reinhardt F (2012). Slug and Sox9 cooperatively determine the mammary stem cell state. Cell.

[49] Van Keymeulen A, Rocha AS, Ousset M, Beck B, Bouvencourt G, Rock J (2011). Distinct stem cells contribute to mammary gland development and maintenance. Nature.

[50] Lapidus RG, Nass SJ, Davidson NE (1998). The loss of estrogen and progesterone receptor gene expression in human breast cancer. J Mammary Gland Biol Neoplasia.

[51] Zvelebil M, Oliemuller E, Gao Q, Wansbury O, Mackay A, Kendrick H (2013). Embryonic mammary signature subsets are activated in *Brca1*^*−/−*^ and basal-like breast cancers. Breast Cancer Res.

[52] Gupta GP, Massagué J (2006). Cancer metastasis: building a framework. Cell.

[53] Du Z, Podsypanina K, Huang S, McGrath A, Toneff MJ, Bogoslovskaia E (2006). Introduction of oncogenes into mammary glands in vivo with an avian retroviral vector initiates and promotes carcinogenesis in mouse models. Proc Natl Acad Sci U S A.

[54] Keller PJ, Arendt LM, Skibinski A, Logvinenko T, Klebba I, Dong S (2012). Defining the cellular precursors to human breast cancer. Proc Natl Acad Sci U S A.

[55] Damonte P, Hodgson JG, Chen JQ, Young LJ, Cardiff RD, Borowsky AD (2008). Mammary carcinoma behavior is programmed in the precancer stem cell. Breast Cancer Res.

[56] Hein SM, Haricharan S, Johnston AN, Toneff MJ, Reddy JP, Dong J, et al. Luminal epithelial cells within the mammary gland can produce basal cells upon oncogenic stress. Oncogene. In press. doi:10.1038/onc.2015.206 10.1038/onc.2015.206 PMC4688047 26096929

[57] Koren S, Reavie L, Couto JP, De Silva D, Stadler MB, Roloff T (2015). *PIK3CA*^H1047R^ induces multipotency and multi-lineage mammary tumours. Nature.

[58] Van Keymeulen A, Lee MY, Ousset M, Brohée S, Rorive S, Giraddi RR (2015). Reactivation of multipotency by oncogenic PIK3CA induces breast tumour heterogeneity. Nature.

[59] Li Y, Welm B, Podsypanina K, Huang S, Chamorro M, Zhang X (2003). Evidence that transgenes encoding components of the Wnt signaling pathway preferentially induce mammary cancers from progenitor cells. Proc Natl Acad Sci U S A.

[60] Santagata S, Thakkar A, Ergonul A, Wang B, Woo T, Hu R (2014). Taxonomy of breast cancer based on normal cell phenotype predicts outcome. J Clin Invest.

[61] Hines WC, Yaswen P, Bissell MJ (2015). Modelling breast cancer requires identification and correction of a critical cell lineage-dependent transduction bias. Nat Commun.

