# Supplementary figures and images for: Transformation of enriched mammary cell populations with polyomavirus middle T antigen influences tumor subtype and metastatic potential
Source: Breast Cancer Res. 2015 Oct 1;17:132. doi: 10.1186/s13058-015-0641-9 (PMC4589945; doi:10.1186/s13058-015-0641-9)

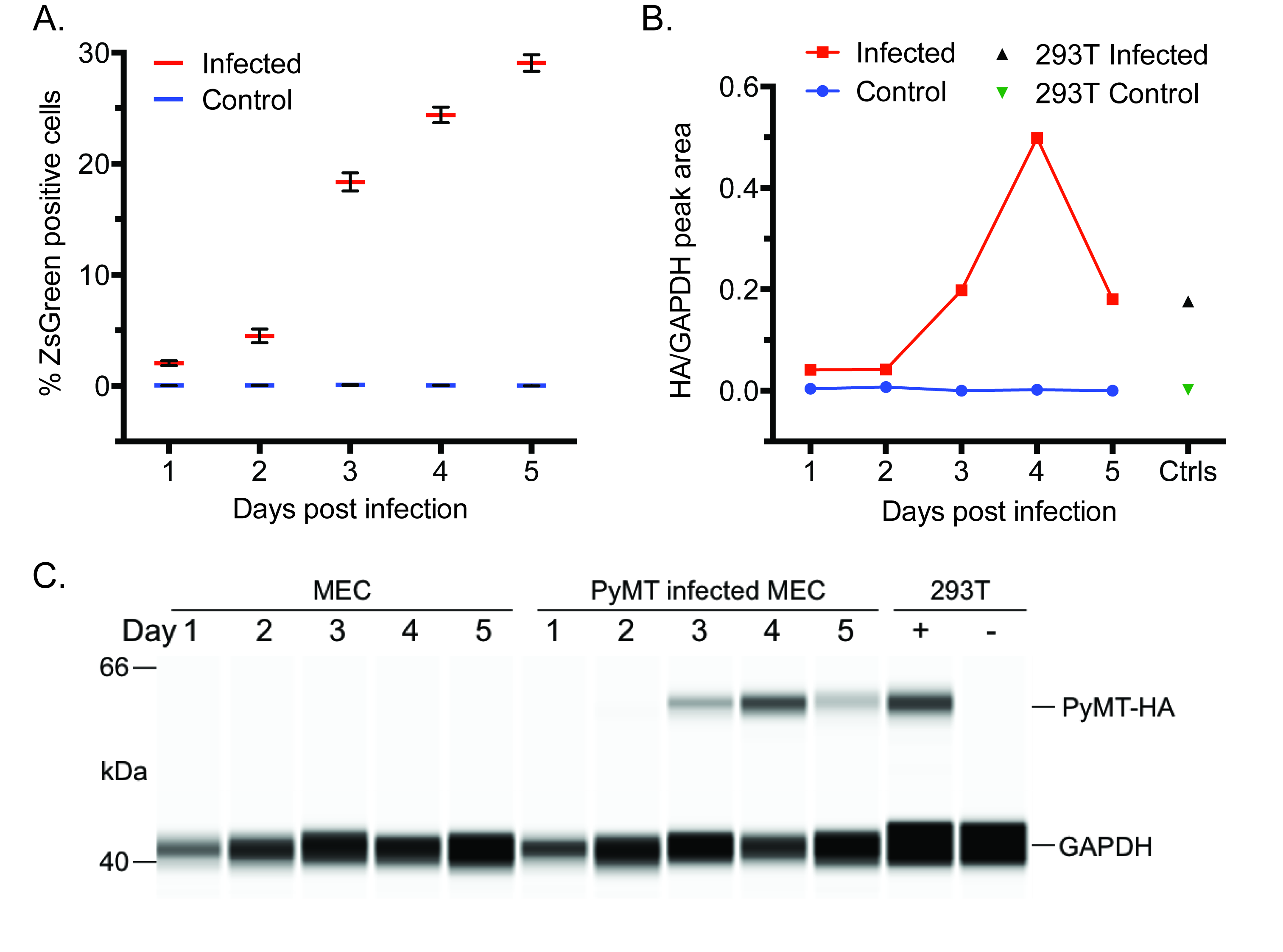

Supplement: Additional file 1: Figure S1. — Postinfection PyMT expression in cultured total MECs. a Flow cytometric analysis of percentage of ZsGreen-positive cells for 5 days post-EF1α-PyMT-ZsGreen infection (10,000 events, mean ± SD, n = 3). b Quantification from simple Western blot analysis of PyMT-HA expression over time in infected MECs and uninfected MEC controls for 5 days following infection. PyMT-HA peak area was normalized to GAPDH peak area. 293 T cells are shown as positive and negative controls for PyMT-HA expression. c Multiplex simple Western blot stained for hemagglutinin (HA) tag and GAPDH. Protein lysates were prepared each day for 5 days following infection and PyMT-HA (60 kDa) with 293 T positive and negative controls. HA tag and GAPDH signals are shown with different exposure times. (TIFF 1824 kb) [file 13058_2015_641_MOESM1_ESM.tif]

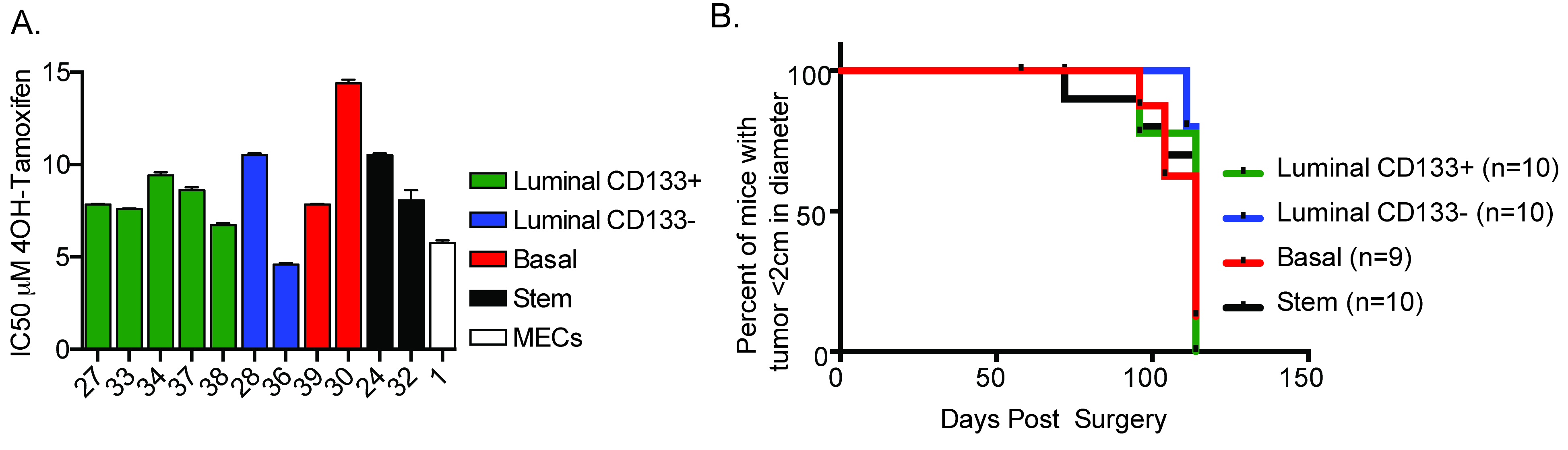

Supplement: Additional file 2: Figure S2. — Hormone independence of EF1α-PyMT-ZsGreen-transduced MEC tumors. a Half-maximal inhibitory concentration values of wild-type MECs and cells isolated from luminal CD133+, luminal CD133−, basal, and stem cell tumors treated for 48 h with 4-hydroxytamoxifen (4-OHT). No significant differences among the viability of tumor cells and normal MECs were observed after 4-OHT treatment (t test). b Assessment of tumor growth response to ovariectomy. Transduced primary cells were transplanted into ovariectomized mice to test if lack of hormone signaling would result in reduced tumor growth from luminal CD133+ cells. No differences in tumor latencies were observed among tumor groups (n = number of mice). (TIFF 1112 kb) [file 13058_2015_641_MOESM2_ESM.tif]

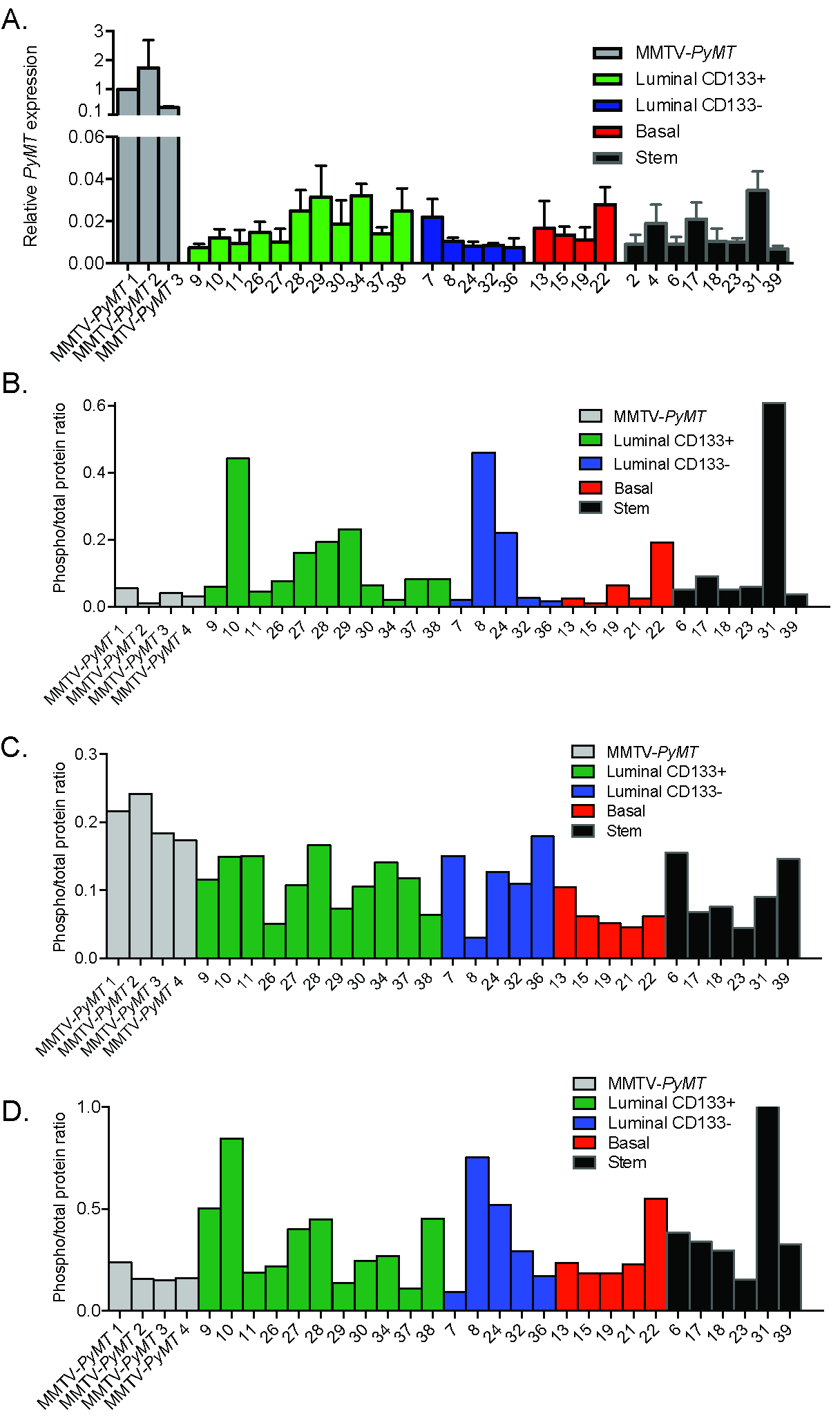

Supplement: Additional file 3: Figure S3. — PyMT expression and activity in tumors. a RT-qPCR quantification of relative PyMT mRNA expression levels in individual MMTV-PyMT, luminal CD133+ cell, luminal CD133− cell, basal cell, and stem cell tumors normalized to the Rplp0 housekeeping gene. Analysis was performed in triplicate for each tumor sample. b Western blot quantification of relative ratio of pAKT to AKT protein expression in individual MMTV-PyMT, luminal CD133+ cell, luminal CD133− cell, basal cell, and stem cell tumors normalized to β-actin. c Western blot quantification of relative ratio of pERK to ERK protein expression in individual MMTV-PyMT, luminal CD133+ cell, luminal CD133− cell, basal cell, and stem cell tumors normalized to β-actin. d Western blot quantification of relative ratio of pSRC to SRC protein expression in individual MMTV-PyMT, luminal CD133+ cell, luminal CD133− cell, basal cell, and stem cell tumors normalized to β-actin. (TIFF 2407 kb) [file 13058_2015_641_MOESM3_ESM.tif]

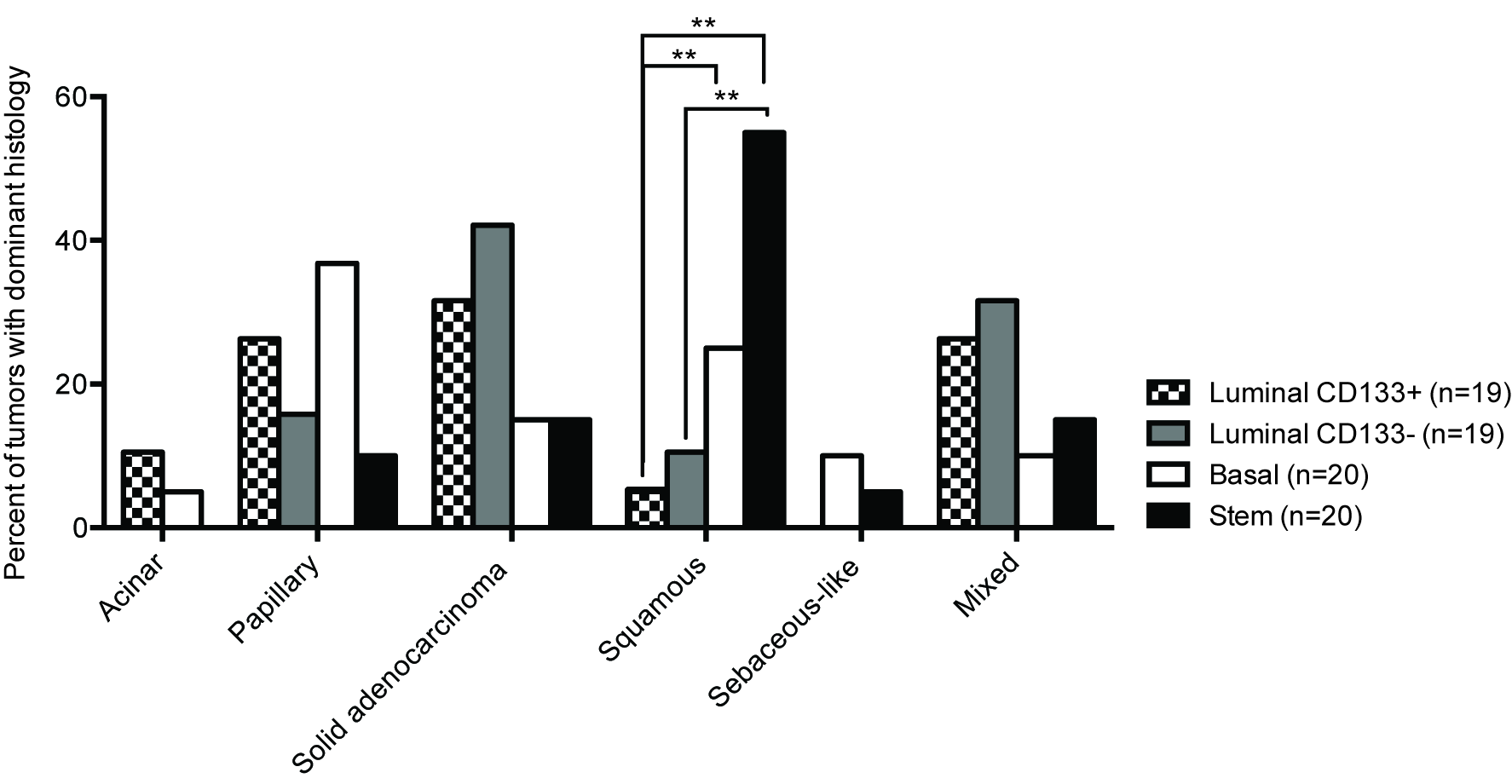

Supplement: Additional file 4: Figure S4. — Percentage of tumors with a dominant histology. Each tumor was classified based on the histological feature representing at least 50 % of the tumor area. Tumors classified as mixed did not have a dominant histological type. Tumors derived from stem cells were significantly more squamous than tumors derived from luminal CD133+ and CD133− luminal cells (two proportion z test, n = number of tumors). (TIFF 833 kb) [file 13058_2015_641_MOESM4_ESM.tif]

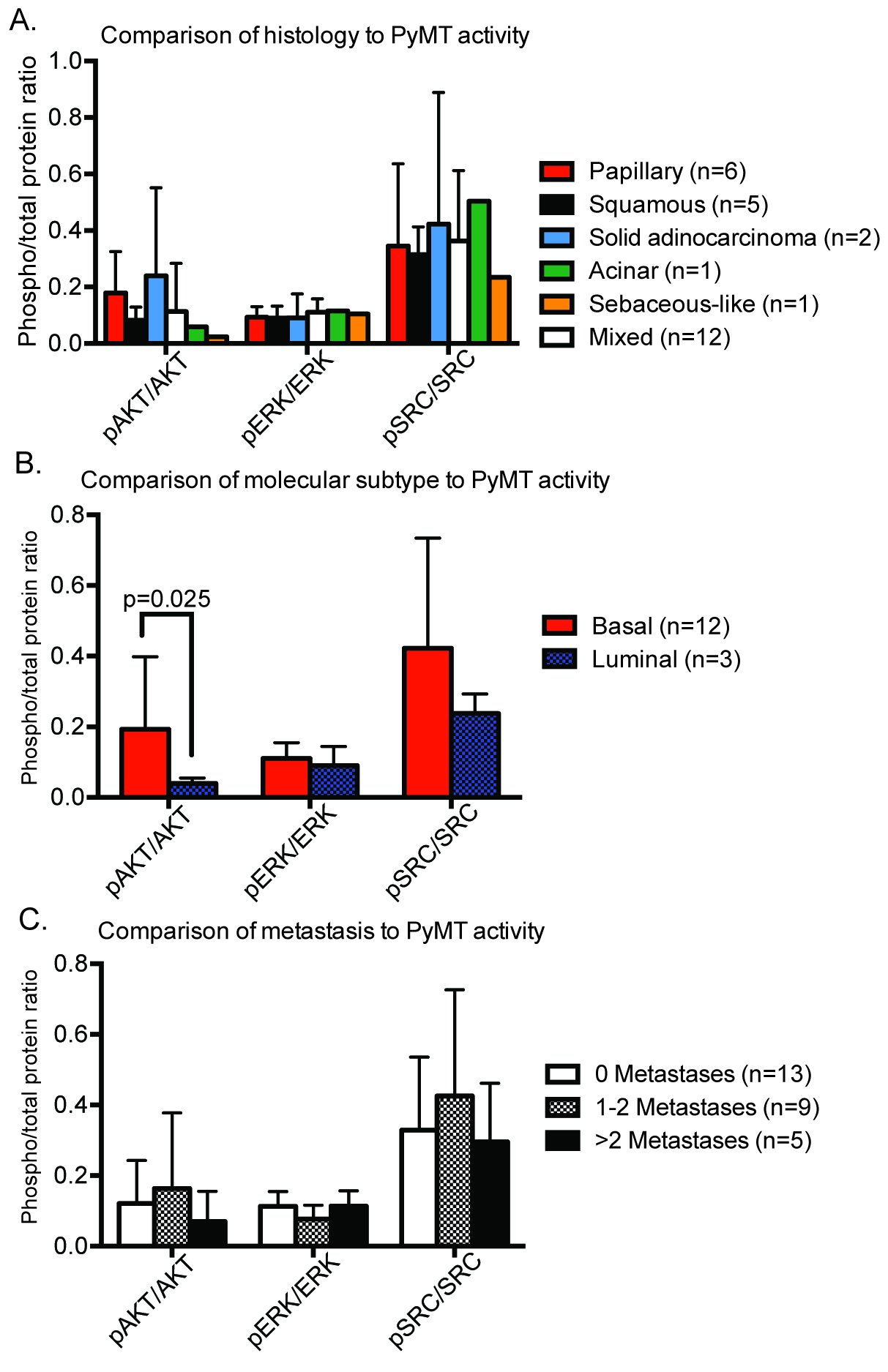

Supplement: Additional file 5: Figure S5. — PyMT activity comparison with tumor histology, molecular subtype, and metastasis. a Western blot quantification of relative ratio of pAKT to AKT, pERK to ERK, and pSRC to SRC protein expression in enriched population tumors segregated based on tumor histology and normalized to β-actin. No significant differences in protein expression were detected among tumor groups (t test, n = number of tumors). b Western blot quantification of relative ratio of pAKT to AKT, pERK to ERK, and pSRC to SRC protein expression in enriched population tumors segregated based on tumor molecular subtype and normalized to β-actin. Molecularly basal tumors exhibited significantly higher levels of pERK/ERK levels than luminal tumors (t test, p value shown, n = number of tumors). c Western blot quantification of relative ratio of pAKT to AKT, pERK to ERK, and pSRC to SRC protein expression in enriched population tumors segregated based on tumor metastasis and normalized to β-actin. No significant differences in protein expression were detected among tumor groups (t test, n = number of tumors). (TIFF 1046 kb) [file 13058_2015_641_MOESM5_ESM.tif]

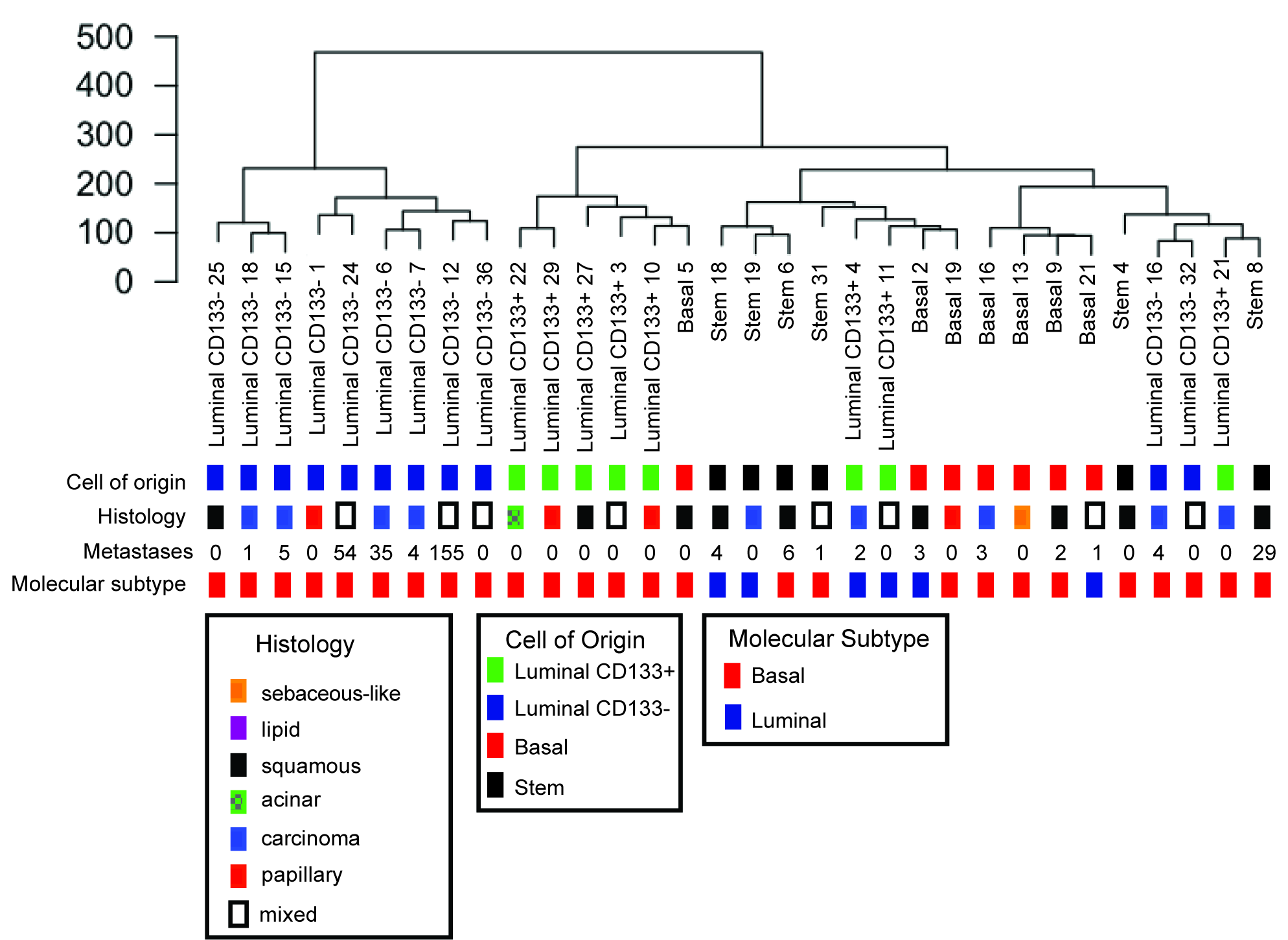

Supplement: Additional file 7: Figure S6. — Unsupervised hierarchical clustering of tumors generated from enriched MEC populations. Tumor cell of origin, histology, metastatic propensity, and molecular subtype are indicated below the dendrogram cluster. (TIFF 1486 kb) [file 13058_2015_641_MOESM7_ESM.tif]

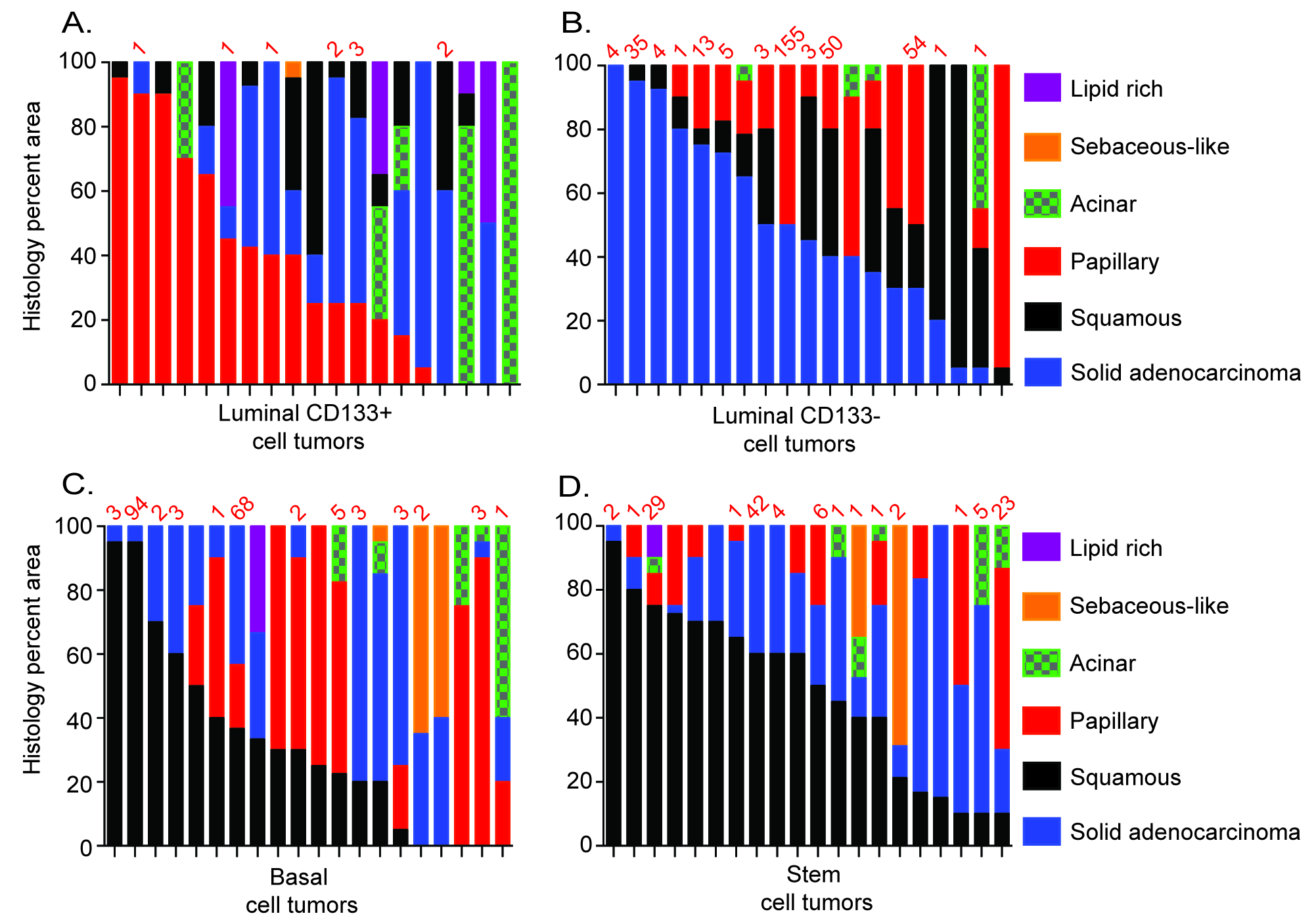

Supplement: Additional file 10: Figure S8. — Number of metastases per tumor histology. Histological area per tumor derived from luminal CD133+ cells (a), luminal CD133− cells (b), basal cells (c), and stem cells (d), as well as the number of metastatic foci detected in the lungs of the mice bearing each of the tumors (shown as red values above each tumor). (TIFF 1562 kb) [file 13058_2015_641_MOESM10_ESM.tif]

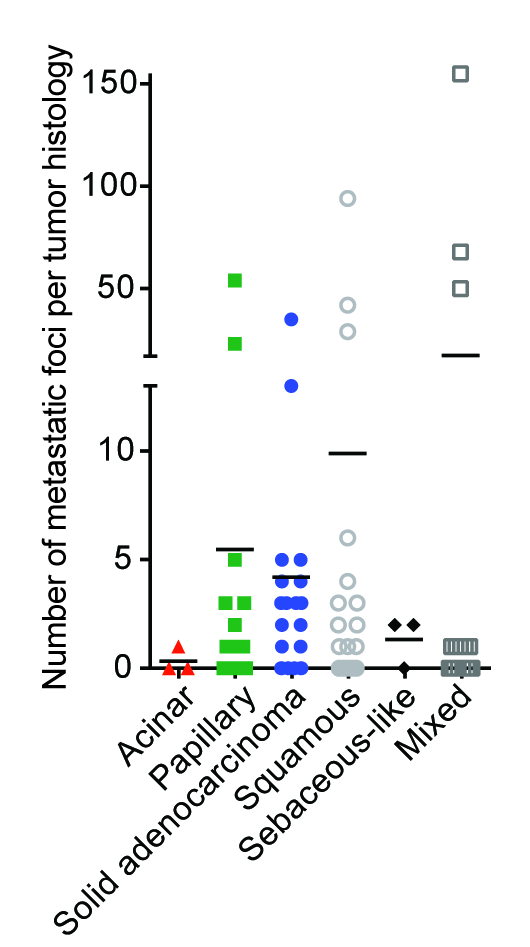

Supplement: Additional file 11: Figure S9. — Quantification of the number of metastases per tumor histology. A histology was assigned to a tumor when the histology comprised at least 50 % of the tumor area. Tumors that had mixed histologies were classified as mixed. No statistically significant differences in the metastatic propensities among histological tumor types were observed (Mann–Whitney U test). (TIFF 654 kb) [file 13058_2015_641_MOESM11_ESM.tif]

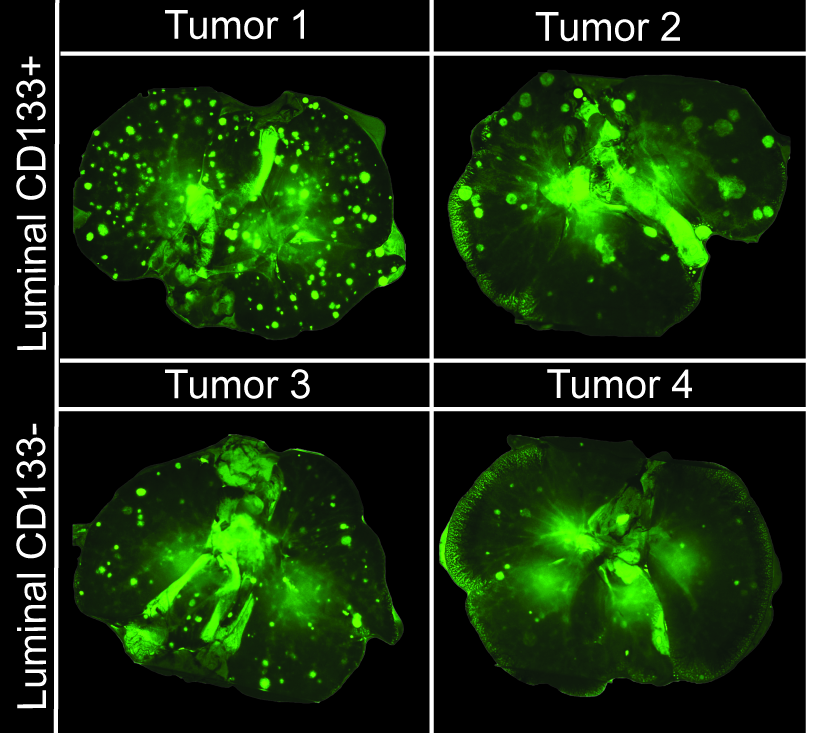

Supplement: Additional file 12: Figure S7. — Enriched MEC populations metastasize to lung. Representative fluorescence images of lungs from mice that received tail vein injections of metastatic luminal CD133+ tumor cells (tumor 1), nonmetastatic luminal CD133+ tumor cells (tumor 2), or metastatic luminal CD133− tumor cells (tumors 3 and 4). Lungs were examined 3 weeks after the tail vein injection. Fluorescent black-and-white lung images were false-colored green in ImageJ software and overlaid on a black background. (TIFF 2284 kb) [file 13058_2015_641_MOESM12_ESM.tif]
