# Supplementary material for: Transformation of enriched mammary cell populations with polyomavirus middle T antigen influences tumor subtype and metastatic potential
Source: Breast Cancer Res. 2015 Oct 1;17:132. doi: 10.1186/s13058-015-0641-9 (PMC4589945; doi:10.1186/s13058-015-0641-9)
Supplement: Additional file 6: Table S1. — Distribution of basal and luminal subtypes. Distribution of basal and luminal tumor subtypes generated by EF1α-PyMT-ZsGreen lentiviral transduction of each sorted cell population as compared with the distribution obtained from EF1α-PyMT-ZsGreen-transduced but unsorted MECs [8], using Fisher’s exact test. (DOCX 14 kb) [file 13058_2015_641_MOESM6_ESM.docx]

**Table S1.** Comparison of the distribution of Basal and Luminal tumor subtypes generated from each cell population with the distribution obtained from unsorted MECs [10], using Fisher’s exact test.

|  | **Sorted** | **Unsorted [10]** |  |
| --- | --- | --- | --- |
| **Transformed population** | **Basal : Luminal** | **Basal : Luminal** | **P-value** |
| Luminal CD133+ | 6 : 2 | 14 : 9 | 0.6757 |
| Luminal CD133- | 11 : 0 | 14 : 9 | 0.0172 |
| Basal | 5 : 2 | 14 : 9 | 1.0000 |
| Stem | 4 : 2 | 14 : 9 | 1.0000 |
